# Towards a quantitative understanding of the MITF-PIAS3-STAT3 connection

**DOI:** 10.1186/1752-0509-6-11

**Published:** 2012-02-08

**Authors:** Josef Thingnes, Timothy J Lavelle, Arne B Gjuvsland, Stig W Omholt, Eivind Hovig

**Affiliations:** 1 Centre for Integrative Genetics (CIGENE), Department of Mathematical Sciences and Technology, Norwegian University of Life Sciences, 1430 Ås, Norway; 2 Centre for Integrative Genetics (CIGENE), Department of Animal and Aquacultural Sciences, Norwegian University of Life Sciences, 1430 Ås, Norway; 3 Department of Tumor Biology, Institute for Cancer Research, The Norwegian Radium Hospital, Montebello, PO Box 4950 Nydalen, 0424 Oslo, Norway; 4 Institute of Medical Informatics, The Norwegian Radium Hospital, Montebello, PO Box 4950 Nydalen, 0424 Oslo, Norway; 5 Institute of Informatics, The Faculty of Mathematics and Natural Sciences, University of Oslo, Postboks 1080, Blindern, 0316 OSLO, Norway

## Abstract

**Background:**

Expression of the two transcription factors microphthalmia-associated transcription factor (MITF) and signal transducer and activator of transcription 3 (STAT3) are tightly connected to cell proliferation and survival, and are important for melanocyte development. The co-regulation of MITF and STAT3 via their binding to a common inhibitor Protein Inhibitor of Activated STAT3 (PIAS3) is intriguing. A better quantitative understanding of this regulation is likely to be important for elucidation of the melanocyte biology.

**Results:**

We present a mathematical model describing the MITF-PIAS3-STAT3 signalling network. A default parameter set was developed, partly informed by the literature and partly by constraining the model to mimic reported behavioural features of the system. In addition, a set of experiment-specific parameters was derived for each of 28 experiments reported in the literature. The model seems capable of accounting for most of these experiments in terms of observed temporal development of protein amounts and phosphorylation states. Further, the results also suggest that this system possesses some regulatory features yet to be elucidated.

**Conclusions:**

We find that the experimentally observed crosstalk between MITF and STAT3 via PIAS3 in melanocytes is faithfully reproduced in our model, offering mechanistic explanations for this behaviour, as well as providing a scaffold for further studies of MITF signalling in melanoma.

## Background

The melanocytes are skin cells of neural crest origin that constitute 5% - 20% of the basal layer of human epidermis [1-6]. The cell type is responsible for the melanin pigment production and thus the colour patterning of skin and hair in mammals. Melanoma, a cancer originating in melanocytes, is in its later stages notoriously resistant to treatment, and although good prognostic markers exist, the understanding of the underlying biology is only slowly forthcoming [7]. While knowledge about each single protein and gene involved in melanocyte development and regulation of homeostasis is important, developing an understanding of the signalling networks connecting the receptors on the surface to the regulating effect on gene transcription in the nucleus appears crucial in implementing efficient molecular treatment strategies in the dawning era of personalized cancer therapy.

Expression of microphthalmia-associated transcription factor (MITF), the signal transducer and activator of transcription 3 (STAT3), and their co-regulation via protein inhibitor of activated STAT3 (PIAS3), are all tightly connected to cell differentiation, proliferation and survival. MITF is considered to be a master regulatory gene for melanocytes, and has been shown to play important roles in the regulation of genes involved in cell cycle progression, including Bcl-2 and CDK2 [8-10]. MITF is also of clinical significance, as MITF mutations in humans cause Waardenburg syndrome type II [11], and a significant number of malignant melanomas harbour MITF amplifications. MITF has also been proposed to be important for both differentiation of melanocytes and for tumour transformation [12]. MITF has two phosphorylation sites influencing the PIAS3 binding: S73 and S409. These sites are phosphorylated by different kinases in the MAPK pathway, the ERK and RSK, respectively [13,14].

STAT3 is a transcription factor involved in signal transduction pathways that are activated by several extracellular stimuli, including the IL-6 family of cytokines. It is tyrosine phosphorylated by the Janus kinase (JAK) or SRC. The resulting signal mediates cell growth, differentiation, and survival [15-17]. The underlying molecular details have only partly been elucidated [18].

PIAS3 has been identified as an inhibitor of both activated STAT3 and MITF [19-23]. PIAS3 can bind activated STAT3, as well as non-activated MITF in one of its two inactive complexes. The phosphorylation of MITF at S409 results in MITF dissociation from the complex, and more PIAS3 is thereby made available. As a result, more STAT3 is bound in complex with PIAS3 and is thus prevented from binding DNA and activating target genes [22,24,25]. Similarly, expression of constitutively active STAT3 will complex with unbound PIAS3, resulting in less PIAS3 being available for binding to MITF. Consequently, more active MITF is observed [22].

**Figure 1:**
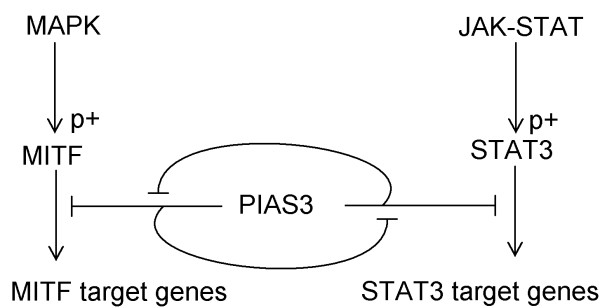
**Overview of the modelled network**. The two transcription factors MITF and STAT3 are phosphorylated by the MAPK-pathway and the JAK-STAT-pathway, respectively. PIAS3 inhibits their activity as transcription factors by binding and forming inactive complexes. The abundance of one transcription factor can sequester PIAS3 to depletion, and eliminate the inhibition of the other transcription factor.

**Figure 2:**
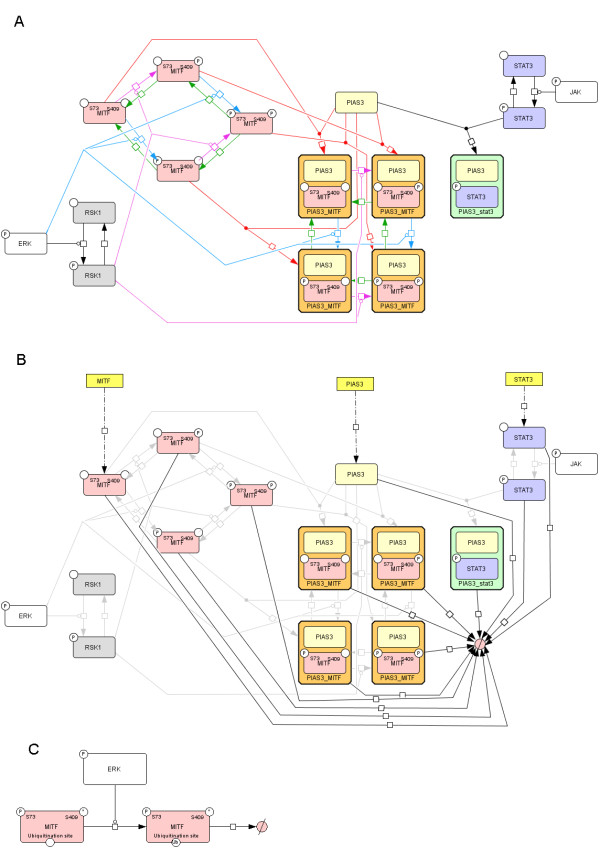
**Graphical representation of the model**. The graphs are represented in Systems Biology Graphical Notation [51] as implemented in CellDesigner [50] version 4.2. A: The diagram shows the different states of each protein (rounded rectangles) and the possible state transitions (arrows). Catalysed transitions are depicted as lines from the catalyst to a circle at the state transition arrow. The model has two input nodes: ERK and JAK (white nodes). ERK is phosphorylating RSK1 (grey nodes). ERK and RSK1 are phosphorylating MITF (red nodes) at S73 (blue lines) and S409 (pink lines), respectively. MITF in different phosphorylation states combines (red lines) with PIAS3 (yellow nodes) forming complexes (orange grouping nodes). On the other side, JAK phosphorylates STAT3 (blue nodes), which is going into complex with PIAS3 when activated (green grouping node). The output nodes of the model are all the free MITF states and the phosphorylated STAT3. Communication between the ERK-RSK-MITF pathway and the JAK-STAT pathway via the competitive binding to free PIAS3 is the topic of this work. B: Production and degradation. The amounts of phosphorylated ERK and JAK are represented by constants. The total amount of RSK1 is constant while the phosphorylation state is dynamically determined. The amounts of MITF, PIAS3 and STAT3 are dynamically determined by the production and degradation of the proteins. The broken arrows from the genes (yellow rectangles) to the proteins represent production, while arrows from the proteins to the degradation node (red filled empty set symbol) represent degradation. C: Ubiquitination of MITF. All S73 phosphorylated MITF can be ubiquitin tagged, in which case it will eventually be degraded. The ubiquitination is catalysed by phosphorylated ERK.

The connection between MITF, STAT3 and PIAS3 (Figure 1) has several interesting features: (1) MITF and STAT3 interacts through binding and sequestration of their common inhibitor PIAS3 [19-22], (2) PIAS3 binds to phosphorylated (activated) STAT3, but disassociates from activated MITF [20] which introduces an asymmetry to the network, (3) MITF has two phosphorylation sites interfering with PIAS3 binding, and all four resulting phosphorylation states have different binding affinities to PIAS3 [20]. We have developed a mathematical model to incorporate quantitative aspects of the system in order to both test if the current conceptions of the system can account for observed results, and to serve as a framework for further studies of this module's interaction with other pathways. See Figure 2 for a graphical representation of the model. This dynamic model of the MITF-PIAS3-STAT3 system was designed to be simple, while still being capable of reproducing the available results. The inputs to the model are the activation events of the two transcription factors MITF and PIAS3. When MITF becomes phosphorylated at S73 and/or S409, the affinity to PIAS3, the degradation rate and the transcriptional activity are altered. On the other hand, STAT3 has only one relevant phosphorylation site, which is phosphorylated by JAK. When STAT3 becomes phosphorylated, the affinity to PIAS3 and the transcriptional activity is altered. As output, the activation levels of the two transcription factors are monitored: For STAT3, this implies the amount of phosphorylated protein, while for MITF it represents a complex feature of the distribution of MITF among the four phosphorylation states.

## Results

### Constraining model behaviour and parameter domains

Default values for the set of core parameters common to all experimental settings were obtained partly directly from the literature and partly by fitting model behaviour to available data. These core parameters describe degradation rate, production rate, binding affinity, and phosphorylation and de-phosphorylation rate (Table 1). For further details on the parameter value deduction, see Methods. We next singled out a representative selection consisting of 28 well-reported experiments addressing various relevant aspects of the MITF-STAT3-PIAS3 system (Table 2), and tested to what degree the model was able to account for the available experimental data. For each experiment, a set of experiment-specific parameters, such as the effect of transfection on production rates or the activation level in growing cells, was specifically incorporated to reflect the experimental setup and conditions. These parameters were specifically set for each experiment. In those cases where the experiments were replicates, the experiment-specific parameters were given identical values. While keeping the core parameters at default values, we manually searched for experiment specific parameter values that minimized the distance between the simulation output and the reported results of the focal lab-experiment. The experiment-specific perturbations of the model are provided in the Methods section. We found that we were able to account reasonably well for 27 of the 28 experiments. Only experiment #26 seems to be beyond explanatory reach by our model framework.

**Table 1:** List of core parameters

No	Parameter	Value	Description
1	*k*_*Mp*73*E*+_	0.00015	MITF phosphorylation on S73, ERK dependent (Mol^-1^min^-1^)

2	*k*_*Mp*73-_	0.03	MITF de-phosphorylation on S73 (min^-1^)

3	*k*_*Mp*73*a*+_	0.025	MITF auto-phosphorylation on S73 (min^-1^)

4	*k*_*Mp*409+_	0.0001	MITF phosphorylation on S409 (Mol^-1^min^-1^)

5	*k*_*Mp*409-_	0.04	MITF de-phosphorylation on S409 (min^-1^)

6	*k_Mass_*	0.01	MITF/PIAS3 association (Mol^-1^min^-1^)

7	*k_Mdiss_*	1	MITF/PIAS3 dissociation (min^-1^)

8	*k*_*Mp*73*ass*_	0.03	MITFp79/PIAS3 association (Mol^-1^min^-1^)

9	*k*_*Mp*73*diss*_	0.5	MITFp79/PIAS3 dissociation (min^-1^)

10	*k*_*Mp*409*ass*_	0.0001	MITFp409/PIAS3 association (Mol^-1^min^-1^)

11	*k*_*Mp*409*diss*_	1	MITFp409/PIAS3 dissociation (min^-1^)

12	*k_Mppass_*	0.01	MITFpp/PIAS3 association (Mol^-1^min^-1^)

13	*k_Mppdiss_*	1	MITFpp/PIAS3 dissociation (min^-1^)

14	*k*_*Sp*+_	0.0002	STAT3 phosphorylation (Mol^-1^min^-1^)

15	*k*_*Sp*-_	0.04	STAT3 de-phosphorylation (min^-1^)

16	*k*_*Spass*_	0.005	STAT3p/PIAS3 association (Mol^-1^min^-1^)

17	*k_Spdiss_*	0.2	STAT3p/PIAS3 dissociation (min^-1^)

18	*p_MITF_*	1	MITF production (Mol min^-1^)

19	*γ_MITF_*	0.0012	MITF degradation (min^-1^)

20	*γ*_*MITFp*73_	0.02	MITFp73 degradation (min^-1^)

21	*γ*_*MITFp*409_	0.01	MITFp409 degradation (min^-1^)

22	*P*_*PLAS*3_	0.262	PIAS3 production (Mol min^-1^)

23	*γ*_*PLAS*3_	0.008	PIAS3 degradation (min^-1^)

24	*P*_*STAT*3_	0.211	STAT3 production (Mol min^-1^)

25	*γ*_*STAT*3_	0.002	STAT3 degradation (min^-1^)

26	*k*_*Rp*+_	0.0004	RSK1 phosphorylation (Mol^-1^min^-1^)

27	*k*_*Rp*_	0.04	RSK1 de-phosphorylation (min^-1^)

28	*ERK_p_*	10	ERKp concentration (Mol)

29	*JAK_p_*	10	JAKp concentration (Mol)

30	*K_u_*	0.0001	Ubiquitination (Mol^-1^min^-1^)

The parameter names follow a naming convention. The rate constants for phosphorylation events is a *k *with a subscript starting with a protein specification (R:RSK1, M:MITF or S:STAT3), followed by a specification of a phosphorylation site (*p*73, *p*409 for MITF and *p *for STAT3) and finalized with a ' + ' for phosphorylation rate constants and ' - ' for de-phosphorylation rate constants. The association and dissociation rate constants is a *k *with a subscript starting with a protein specification followed by a phosphorylation state specification, and finalized with ' *ass *' or ' *diss *' respectively. The name of the parameters denoting the production or degradation rates start with a *p *or a *γ *respectively, and ends with a specification of the protein (and eventually phosphor state). The two constants ERKp and JAKp, represent the amounts of phosphorylated ERK and JAK, respectively. These are altered to emulate signalling through the respective pathways. '*k_u_*' is the ubiquitination rate constant.

**Table 2:** An overview of the biological experiments

Description		Reference
1.	Temporal development of ERK and RSK1 kinase activity	[14]Figure 5B

2.	Distribution between MITF phosphorylation before stimulation	[14]Figure 1 and 2

3.	Temporal development of MITF phosphorylation states after stimulation	[14]Figure 1 and 2

4.	Temporal development of MITF degradation	[14]Figure 1 and 2

5.	MITF activity in response to PIAS3 transfection	[19]Figure 6

6.	Amount of MITF-PIAS3 complex in response to activation	[20] Figure 2A

7.	Amount of MITF-PIAS3 complex in response to activation with transfected MITF and PIAS3	[20] Figure 2B

8.	MITF activity in response to transfection of constitutively active RSK1	[20] Figure 3A

9.	MITF activity in response to PIAS3	[20] Figure 3B, left, black

10.	MITF activity in response to transfection of PIAS3 and active RSK1	[20] Figure 3B, left, grey

11.	Activity of a MITF-S409A mutant in response to PIAS3	[20] Figure 3B, right, black

12.	Activity of a MITF-S409A mutant in response to transfection of PIAS3 and active RSK1	[20] Figure 3B, right, grey

13.	Activity of MITF in response to transfection of PIAS3	[20] Figure 6

14.	Activity of MITF-S73D mutant in response to transfection of PIAS3	[20] Figure 6

15.	Activity of MITF-S409D mutant in response to transfection of PIAS3	[20] Figure 6

16.	Activity of MITF-S73/409D mutant in response to transfection of PIAS3	[20] Figure 6

17.	MITF-PIAS3 association in response to activation	[22] Figure 3A

18.	S409A mutated MITF-PIAS3 association in response to activation	[22] Figure 3A

19.	STAT3 activity in response to activation	[22] Figure 3B

20.	STAT3 activity in response to activation with transfected PIAS3	[22] Figure 3B

21.	STAT3 activity in response to activation with transfected PIAS3 and small amount of MITF	[22] Figure 3B

22.	STAT3 activity in response to activation with transfected PIAS3 and large amount of MITF	[22] Figure 3B

23.	STAT3 activity in response to activation with transfected PIAS3 and small amount of S-409A mutated MITF	[22] Figure 3B

24.	STAT3 activity in response to activation with transfected PIAS3 and large amount of S409A mutated MITF	[22] Figure 3B

25.	MITF activity in response to transfection of Y705F mutated STAT3 and STAT3-C	[22] Figure 4B

26.	MITF and STAT3 activity in response to activation compared between wild type and mutated MITF (unable to bind PIAS3)	[22] Figure 6D and E

27.	STAT3 activity in response to transfection of MITF or S409D MITF mutation	[22] Figure 7C

28.	MITF activity in response to transfection of STAT3-C	[22] Figure 7D

The numbers assigned to each experiment in the table is used to identify the experiment throughout this paper. For each experiment the reference and the figure number within the reference are provided.

### Robustness of fit between model results and experimental data

**Figure 3:**
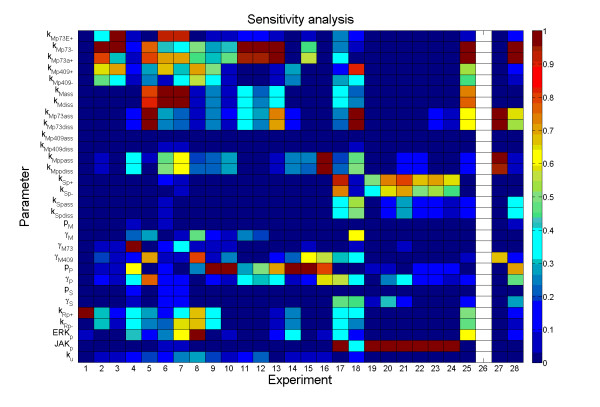
**Parameter sensitivity for all experiments**. Sensitivity for all experiments to perturbations in each parameter after permutations to remove clearly insensitive parameters (no parameters passed the permutation threshold for experiment #26) is shown. Colour indicates the normalized sensitivity measure $\frac{s_{i,j}}{\underset{j}{\max(s_{i,j})}}$ Each experiment is insensitive to perturbations in the dark blue parameters, while highly sensitive to the brown parameters.

We next investigated how sensitive the obtained fit between the experimental data and the model results was with respect to variation in the core parameter values, in the 27 successful cases examined. While keeping the experiment-specific parameters unchanged, 10^6 ^sets of the core parameters were sampled uniformly on a logarithmic scale from a hypercube centred at the default parameter values and within the range 0.5-2.0× of each default value. For all sampled parameter sets, we simulated the 27 successful experiments in Table 2 and recorded success or failure accordingly. By analysing the 30 × 10^6 ^parameter matrix and the corresponding 28 × 10^6 ^result matrix we studied the sensitivity of the success rate of each experiment to variation in a given parameter by computing success rates in bins of sorted parameter sets, and used the sum of absolute deviations from the overall success rate to range the parameters according to sensitivity (Figure 3). Each experiment tested different features of the system, which is reflected in the parameter sensitivity. Some experiments are sensitive only to one parameter. For example experiment #1, which investigated the temporal development of ERK and RSK1 kinase activities, and experiment #14, which investigated the transcriptional activity of the MITF-S73D mutant in response to transfection of PIAS3 were both sensitive to a single parameter, the RSK1 phosphorylation rate constant *k*_*Rp*+ _and the PIAS3 production rate *P*_*PLAS*3_, respectively. On the other hand, experiment #17, which investigated the MITF-PIAS3 association in response to activation, and experiment #18, which investigated S409A mutated MITF-PIAS3 association in response to activation were both sensitive to more than half the parameters. Further, it was observed that the experiments that dealt only with MITF and the MITF-PIAS3 connection, and not with STAT3 (experiments #2 - #16), were only affected by the parameters regarding MITF. On the other hand, experiments #19 to #24 were sensitive only to perturbations of the STAT3 phosphorylation and de-phosphorylation constants, as well as the level of phosphorylated JAK. Notably, three parameters did not affect the results of any experiment: *k*_*Mp*409*ass*_, *k*_*Mp*409*diss*_, and *P_MITF_*. In addition, three parameters had only weak effects: *P*_*STAT*3_, *γ*_*STAT*3_and *k_u_*.

### Interpretation and qualification of selected experiments

**Figure 4:**
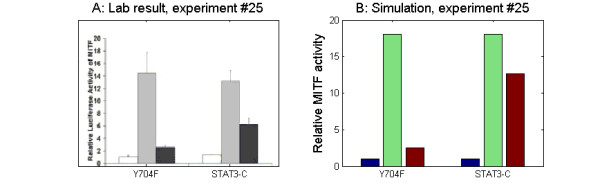
**Simulations and lab results experiment #25**. A: Reprint of figure 4B in [22] (License agreement number 2838800627526): NIH 3T3 cells were co-transfected with the luciferase reporter under the control of the mMCP6 promoter, MITF, PIAS3, and STAT3 mutants (STAT3-C and Y705F-STAT3). B: Simulation of the same experiment (Experiment 25 in Table 2). The white bars in the reprint and the blue bars in the simulation results represent the MITF activity in cells transfected with only STAT3, the grey bars in the reprint and the green bars in the simulation results represent the MITF activity in cells transfected with STAT3 and MITF, and the black bars in the reprint and the red bars in the simulation results represents the MITF activity in cells transfected with STAT3, MITF and PIAS3.

Most of the experiment-specific simulations suggest that the main underlying biological mechanisms seem to be captured by the model. As an example, the result of experiment #25 is plotted alongside the original figure from [22] in Figure 4. The temporal development for all variables for all experiment simulations can be found in Additional file 1 - simulationFigures.zip. In the following, we consider only those experiments in need of further interpretation and qualification.

**Figure 5:**
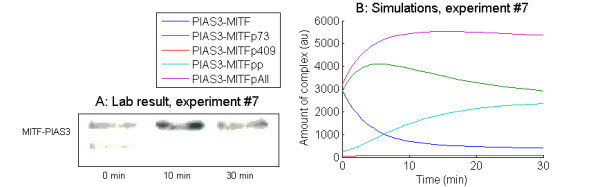
**Simulations and lab results experiment #7**. A: Reprint of Figure 2B in [20] (License agreement number 2773700432120): Kinetics of MITF and PIAS3 association in TPA-activated BL6-B16 cells. Coimmunoprecipitation (IP) of PIAS3 with MITF at defined time points in TPA-activated BL6-B16 cells. The cells were transfected with pcDNA-MITF and pcDNAPIAS3. The blots were probed with anti-MITF antibodies and then reprobed with anti-PIAS3 antibody. Phosphorylation states represented by the upper band are unknown, the lower band is anticipated to represent un-phosphorylated MITF. B: Simulation of the same experiment (experiment #7 in Table 2). The temporal development of the amount of MITF-PIAS3 complex is plotted for all phosphorylation states of MITF, as well as for the sum of the phosphorylated states (PIAS3-MITFpAll).

In experiment #7, the MITF-PIAS3 association after activation was investigated. BL6-B16 melanoma cells were co-transfected with MITF and PIAS3. After 48 hours, the cells were treated with tetradecanoyl phorbol acetate (TPA) for 30 minutes. The amount of MITF-PIAS3 complex was measured before, and after 10 and 30 minutes of TPA treatment (Figure 5A). Two different interpretations of the two bands representing MITF have been proposed. In all cases, the lower band is considered as representing un-phosphorylated MITF, while the upper band may represent all phosphorylated states, or alternatively only S73 phosphorylated MITF [14,26]. If we assume that the upper band corresponds to all phosphorylated MITF-PIAS3 complexes (which is in accordance with what is assumed about the bands representing MITF in [14]), we can predict the temporal development of the distribution among the different phosphorylation states. In our simulations (Figure 5B), the amount of un-phosphorylated MITF-PIAS3 complex decreases rapidly, which is in accordance with the lower band in Figure 5A. The sum of all the phosphorylated states is high after 10 minutes, and is falling again after 30 min, which is in accordance with the higher band in Figure 5I. The distribution among the phosphorylated states can thus be viewed as a prediction. However, it has been suggested that the upper band represents only S73 phosphorylated MITF [26], which is also supported by our model. The S73 phosphorylated complex is also high after 10 minutes, and falls again after 30 minutes. If this is the case, the temporal development of the other phosphorylated complexes (MITF-PIAS3p409, MITF-PIAS3pp) can be viewed as predictions of undetected MITF-PIAS3 phosphorylation states.

**Figure 6:**
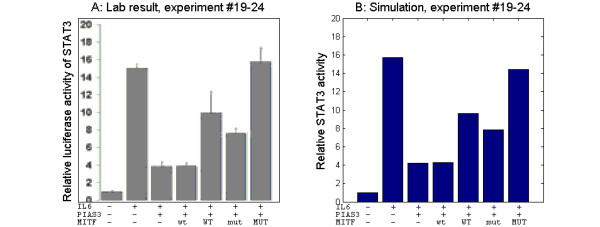
**Simulations and lab results experiments #19 - #24**. A: Reprint of Figure 3B in [22] (License agreement number 2838800627526): NIH 3T3 cells were co-transfected with STAT3, PIAS3 and with different doses of MITF (wild type [WT] or S409A [MUT] (capital letter denoting the greater dose)). Cells were triggered with IL-6/IL-6R for 6 h. B: Simulation of the same experiment (experiments 19-24 in Table 2).

In experiments #19 - 24, the transcriptional activity of STAT3 was measured in NIH 3T3 fibroblasts that were co-transfected with STAT3, PIAS3, and with different doses (0.05 μg, 0.1 μg) of MITF (wild type [WT] or S409A [MUT]). After 48 hours of incubation, the cells were activated with IL6/IL6R for 6 h. Transcriptional activity of STAT3 was measured, and the mean ± the SEM of three experiments is shown in Figure 6A. The simulations of these experiments, which were performed with equal experiment-specific parameters, captured the reported results well (Figure 6B). However, there is a striking, but un-noticed result both in the lab experiment and the simulation in that there is no effect of adding the smaller amount of wtMITF, while there is a rather big effect of adding the greater amount of wtMITF. Because of this similarity in the nonlinearity between the model simulation and the data, an inspection of the model and the simulation results may provide insight to the underlying mechanism. During the incubation time, when the transfected genes are being transcribed and translated to their respective proteins, complexes will be formed continuously. When MITF is added, the PIAS3-MITF complex will form. The sequestering of PIAS3 is not only making it unavailable for STAT3 inhibition, but also rescues PIAS3 from degradation. In the case where the smaller amount of MITF was added, these two effects would have cancelled each other out, while in the case where the greater amount of MITF was added, all PIAS3 would be sequestered, resulting in a saturated rescuing effect.

**Figure 7:**
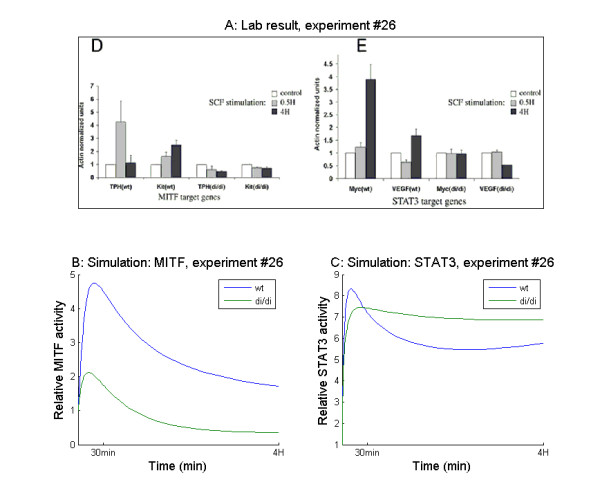
**Simulations and lab results experiment #26**. A: Reprint of figure 6D and E from [22] (License agreement number 2838800627526): Quantitative reverse transcription-PCR analysis of MITF target genes (c-Kit and TPH) and STAT3 target genes (c-Myc and VEGF) from BMMC activated for 0.5 or 4h with SCF derived from either wild-type or MITF^di/di ^mice. (B and C) Simulations of the same experiment (experiment #26 in Table 2). Note that the di/di mutation blocks the activation of both MITF and STAT3 in the reprinted lab result while in the simulation STAT3 activity is not affected.

In experiment #26, a comparison was performed between MITF wild type and a mutant that was transcriptionally active, but unable to bind PIAS3. Transcriptional activity was measured by rtPCR on the mRNA of MITF and STAT3 target genes at 30 minutes and at 4 hours after activation. When the cells were transfected with wild type MITF, elevated transcriptional activity was observed for both transcription factors after activation. However, when the cells were transfected with the MITF mutant, neither MITF nor STAT3 displayed elevated transcriptional activity (Figure 7A). The model was able to mimic the change in transcriptional activity of MITF in reaction to activation for both wild type and mutated MITF (Figure 7B), while the transcriptional activity of STAT3 was not in accordance with the experimental data. In the simulation of this experiment, the transcriptional activity of STAT3 increased in response to activation, regardless of MITF mutation status (Figure 7C). In the lab experiment, this increased transcriptional activity was only seen in the cells with wild type MITF and not in the cells with mutated MITF. The authors of the original paper conclude that PIAS3, being unable to bind the mutated MITF, is accessible for binding and thus inhibition of STAT3. In the model simulations, there was indeed more PIAS3 available in the case with mutated MITF, which causes the lower peak in the level of phosphorylated STAT3 in this case (Figure 7C). However, due to MITF degradation, similar levels of PIAS3 becomes available also in the case of wild type MITF, leading to no difference in level of free PIAS3 to inhibit phosphorylated STAT3 activity. Thus, according to our results, the proposed mechanism is not a plausible explanation of the observed data.

## Discussion

The focal melanocyte regulatory module may reflect the need for co-regulation of two transcription factors. It seems to provide one type of solution for obtaining different co-regulation effects on different time scales, from the immediate effects on the activity of one transcription factor upon activation of the other, to the long term effect of sequestering and later release. Because this module has components working on many different time scales, the history leading up to an event may be determinative. This may be of importance when interpreting results from experiments that operate on different time scales, such as transfection of constitutively active kinases compared to activation of pre-existing kinases.

### The cross talk is going mostly one way

The asymmetry of the network, introduced by PIAS3 binding to activated STAT3, while most efficiently binding to non-activated MITF, yields a particular regulatory effect. We observe that in the model, STAT3 is more affected by the activation of MITF than MITF is affected by the activation of STAT3. This phenomenon, which is in accordance with earlier observations [19,20], can be elucidated from some features of the model. In the resting cell, PIAS3 is to a larger extent bound to un-phosphorylated MITF and MITFp73, which are the major phosphorylation states in the resting cell. On the other hand, STAT3 only binds PIAS3 when activated. Thus, even if both transcription factors are able to alter the amount of free PIAS3, only STAT3 is affected by it: Activated MITF does not interact with PIAS3, and is therefore unaffected by the amount of free PIAS3. STAT3, on the other hand, binds PIAS3 only in activated state, and the strength of the signal is therefore highly affected by the level of free PIAS3. This is a mechanistic explanation for the observation that STAT3 does not associate with PIAS3 that is already associated with MITF [19].

### This model cannot explain the result of experiment #26

Our results indicate that it is not possible to mimic experiment 26 with this model. This finding contradicts the mechanism suggested in [22] as an explanation for the observed data. In our opinion, these observations must either be caused by un-expected properties in the MITF mutant, and/or by un-modelled connections in the network. The latter is supported by the fact that the module modelled here is an abstraction of a small part of a vastly complex regulatory network. Mechanisms not accounted for in the present model include regulation of MITF [10,27,28] and upstream co-regulators in the JAK-STAT [29] and the SRC-STAT pathways.

### The sensitivity of simulation results to parameter value perturbations

In the sensitivity analysis the model parameters were perturbed in a range from half to double their default value. Some of the parameters exposed negligible effect on the simulation results under these perturbations. For instance there are no experiments that show high sensitivity to the association and dissociation constants *k*_*Mp*409*ass*_, *k*_*Mp*409*diss*_. A possible explanation for this is that the affinity of S409 phosphorylated MITF to PIAS3 is so low that PIAS3-MITFp409 is rare compared to the other PIAS3-MITF complexes, and modest perturbations of the association and dissociation constants does not change this situation. The MITF and STAT3 production rates (*P_MITF _*and*P*_*STAT*3_) are affecting the total amount of protein, but still none of the experiments are particularly sensitive to these parameters. The reason for this seems to be that the relative response measured in the experiments is not affected much by this total amount.

### The broader context

In the skin, the major contributors to melanocyte signalling are dermal fibroblasts and epidermal keratinocytes [30,31]. For MITF, the major regulatory pathways are thought to be the TGF-β and the POMC derivatives ACTH and α-MSH signalling pathways [32]. The TGF-β pathway, which is often dysregulated in melanoma [33,34], down-regulates MITF, while the α-MSH pathway up-regulates MITF expression [28,35,36]. The up-regulation occurs via α-MSH binding and activation of MC1R, which leads to cAMP/PKA activation and activation of MAPK [36,37]. Thus, the effect of the MAPK pathway on MITF is complex, including up-regulation, activation and tagging for degradation. For STAT3, in addition to the IL-6 cytokine family, growth factors such as EGF, PDGF and KITL have been reported to activate STAT3 [38-40]. Here, KITL is of special interest in melanocyte biology. In addition to being essential for melanocyte development, it has recently been reported that the hereto unidentified coupling of MC1R activation to MAPK proceeds via MC1R-coupled KIT activation of MAPK [41]. With a focus on developing potential therapies toward melanoma, both MITF and STAT3 have been implicated in neoplastic progression. MITF has been classified as a *bona fide *context dependent oncogene [12]. STAT3 has been implicated in neoplastic progression with an increased contribution especially in metastatic melanoma [42]. Strikingly, no spontaneous or inherited mutations have been isolated [16]. Targeting STAT3 is, however, feasible from both upstream and downstream elements. In addition to being a melanoma oncogene, depletion of MITF has been reported to induce arrest, senescence and cell death in melanoma identifying it as a potential target for therapy [43,44]. Indeed, a PIAS3 based peptide has been developed to target both MITF and STAT3 via a PIAS3 23-aa mimic [21,23].

### Applicability of the Model

With regards to MITF and STAT3 and their roles in melanocyte biology, it is intriguing that α-MSH signalling is somewhat equivalent to KITL signalling with the additional activation of the cAMP/PKA axis. This leads to an increase in MITF production and dual phosphorylation of both MITF and STAT3. Likewise, α-MSH signalling in melanoma predisposing MC1R RHC (red hair colour) mutant backgrounds, which does not elevate cAMP, would alone be equivalent to KITL signalling in a wild type background with regards to MITF and STAT3, the outcome being STAT3 phosphorylation and MITF activation and subsequent depletion. With this in mind, the emergence of MITF's role in melanoma and its high frequency of duplication, it seems contradictory that MC1R mutants predispose individuals to this disease. It will be interesting to see if compensatory mutations in cAMP elevating factors such as G protein-coupled receptors (*e.g*. the histamine receptor H2) are found to be necessary for progression and if so to model the outcomes [45-47].

Special conditions like JAK-STAT3 activation duration due to SOCS feedback inhibition versus SRC-STAT3 activation, which is not influenced by SOCS [16], may be simulated through the model. In addition, other relevant factors such as NF-κ-B, which is also regulated by PIAS3 binding [48], may be added by incorporating an NF-κ-B module. Another interesting module would, for example, be GM-CSF/KITL or GM-CSF/α-MSH signalling. GM-CSF activates MAPK, but has been reported to inhibit KIT signalling via direct binding of CSF2RA to KIT [49].

With this model at hand, more detailed issues in this network can be addressed. The previously posed question on which phosphorylation states of MITF are represented by the upper band interpreted to be phosphorylated MITF in Western blot analysis [13,14], is such an issue. With the ability to test different hypothesis in simulations, both the experiment design and the interpretation of the experimental results could benefit.

Further work could include refinement of various parts of the model by integrated wet-lab modelling efforts. The three parts of the model that could benefit the most from structural enhancement, are the following: (i) The mapping function from the MITF phosphorylation states to the MITF transcriptional activity could be represented on a higher resolution level. This is needed for the model to address quantitative data with absolute values. (ii) The production regime of MITF, PIAS3, STAT3 and RSK1 could be represented by both transcription and translation. (iii) The enzymatic equations could be represented by Michaelis-Menten kinetics. All these structural changes will introduce more state variables and model parameters, thus nothing would be gained by these efforts without the generation of accurate quantitative data to pin down both the model structure and the parameter values.

## Conclusions

In this work, we have provided a mathematical model of the MITF-PIAS3-STAT3 network and have mimicked a representative selection of lab experiments that explore the features of this network. The analyses of this model have revealed explanations to the observed phenomena, as well as recommended reconsideration of previously proposed explanations. This model provides a framework for further investigation of this interesting crosstalk, and can be used as a tool for experimental design and as starting point for further modelling efforts.

## Methods

### Model description

We have developed an ordinary differential equation (ODE) model of the MITF-PIAS3-STAT3 system. The graphical representation of the model given in Figure 2 is represented in Systems Biology Graphical Notation as implemented in CellDesigner [50]. The ODE-model was implemented in MATLAB (Additional file 2 - MATLABcode.zip). In the following the model is presented in terms of the chemical reaction equations and the ordinary differential equations. All protein amounts represented by the state variables in the model are presented in an arbitrary unit (au).

The amount of phosphorylated ERK is represented by a constant (a MAPK signal is represented by an altering of this constant). This kinase is involved in two processes; phosphorylation of RSK1 (1) and phosphorylation of MITF at S73 (2).

(1) $$RSK1\overset{\left[ERK_{p}\right]k_{Rp+}}{\underset{k_{Rp-}}{⇌}}RSK1_{p}$$

In all the equations the subscript *p *after the protein denotes phosphorylated form. The phosphorylation and de-phosphorylation rate constants are represented by *k_Rp+ _*and *k_Rp- _*respectively.

(2) $$\begin{array}{c} I)\;MITF\overset{\left[ERK_{p}\right]k_{Mp73E+}}{\underset{k_{Mp73-}}{⇌}}MITF_{p73}\\ II)\;MITF_{p409}\overset{\left[ERK_{p}\right]k_{Mp73E+}}{\underset{k_{Mp73-}}{⇌}}MITF_{pp}\\ III)PIAS3\_MITF\overset{\left[ERK_{p}\right]k_{Mp73E+}}{\underset{k_{Mp73-}}{⇌}}PIAS3\_MITF_{p73}\\ IV)\;PIAS3\_MITF_{p409}\overset{\left[ERK_{p}\right]k_{Mp73E+}}{\underset{k_{Mp73-}}{⇌}}PIAS3\_MITF_{pp} \end{array}$$

All the equations in (2) represent *ERK_p _*catalyzed phosphorylation of MITF at S73. Subscripts after MITF denote phosphorylation state (un-phosphorylated, S73 phosphorylated, S409 phosphorylated and phosphorylated at both sites). Complexes are represented by underscore separated protein abbreviations. Note that the same phosphorylation (*k*_*Mp*73E+_) and de-phosphorylation (*k*_*Mp*73-_) rate constants are used in all four equations. In addition MITF is auto-phosphorylated at S73 governed by a separate rate constant, *k*_*Mp*73*a*+_:

(3) $$\begin{array}{c} I)\;MITF\overset{k_{Mp73a+}}{\to }MITF_{p73}\\ II)\;MITF_{p409}\overset{k_{Mp73a+}}{\to }MITF_{pp}\\ III)PIAS3\_MITF\overset{k_{Mp73a+}}{\to }PIAS3\_MITF_{p73}\\ IV)\;PIAS3\_MITF_{p409}\overset{k_{Mp73a+}}{\to }PIAS3\_MITF_{pp} \end{array}$$

Further, the phosphorylation of MITF at S409 is catalyzed by the phosphorylated RSK1:

(4) $$\begin{array}{c} I)\;MITF\overset{\left[RSK1_{p}\right]k_{Mp409+}}{\underset{k_{Mp409-}}{⇌}}MITF_{p409}\\ II)\;MITF_{p73}\overset{{\left[RSK1_{p}\right]}_{kMp409+}}{\underset{k_{Mp409-}}{⇌}}MITF_{pp}\\ III)PIAS3\_MITF\overset{\left[RSK1_{p}\right]k_{Mp409+}}{\underset{k_{Mp409-}}{⇌}}PIAS3\_MITF_{p409}\\ IV)\;PIAS3\_MITF_{p73}\overset{\left[RSK1_{p}\right]k_{Mp409+}}{\underset{k_{Mp409-}}{⇌}}PIAS3\_MITF_{pp} \end{array}$$

The four different phosphorylation states have different association and dissociation rate constants:

(5) $$MITF+PIAS3\overset{k_{Mass}}{\underset{k_{Mdiss}}{⇌}}PIAS3\text{\_}MITF$$

(6) $$MITF_{p73}+PIAS3\overset{k_{Mp73ass}}{\underset{k_{Mp73diss}}{⇌}}PIAS3\text{\_}MITF_{p73}$$

(7) $$MITF_{p409}+PIAS3\overset{k_{Mp409ass}}{\underset{k_{Mp409diss}}{⇌}}PIAS3\text{\_}MITF_{p409}$$

(8) $$MITF_{pp}+PIAS3\overset{k_{Mppass}}{\underset{k_{Mppdiss}}{⇌}}PIAS3\text{\_}MITF_{pp}$$

STAT3 is also binding PIAS3:

(9) $$STAT3_{p}+PIAS3\overset{k_{Spass}}{\underset{k_{Spdiss}}{⇌}}PIAS3\text{\_}STAT3_{p}$$

And finally the phosphorylation of STAT3 is catalyzed by phosphorylated JAK (which is represented by a constant):

(10) $$STAT3\overset{\left[JAK_{p}\right]k_{Sp+}}{\underset{k_{Sp-}}{⇌}}STAT3_{p}$$

A signal propagating through the JAK-STAT3-pathway is represented in the model simulations by an elevation of the constant representing the amount of phosphorylated JAK.

These chemical reactions were translated into an ODE model by anticipating simple mass action reaction kinetics. See Table 2 for a comprehensive list of parameters. Equation (11), describing the dynamics of unbound and un-phosphorylated MITF, is provided in detail below. One differential equation for each phosphorylation and complex state of each protein is given. These equations are in the same way determined from the chemical equations and are therefore only commented when features differ.

In this model, MITF can be present in 8 different states and is therefore represented by 8 differential equations, each containing parts reflecting the chemical reactions involving that particular state.

(11) $$\begin{array}{c} \overset{•}{\left[MITF\right]}=p_{MITF}\\ -k_{Mp73E+}\left[MITF\right]\left[ERK_{p}\right]-k_{Mp73a+}\left[MITF\right]\\ +k_{Mp73-}\left[MITF_{p73}\right]\\ -k_{Mp409+}\left[MITF\right]\left[RSK1_{p}\right]+k_{Mp409-}\left[MITF_{p409}\right]\\ -k_{Mass}\left[MITF\right]\left[PIAS3\right]+k_{Mdiss}\left[PIAS3\_MITF\right]\\ -\gamma_{MITF}\left[MITF\right] \end{array}$$

In differential equation (11), where the rate of change of un-phosphorylated and un-bound MITF is described, *P_MITF _*is the production rate, *k*_*Mp*73*E*+_[*MITF*][*ERK_p_*] is the rate of MITF leaving this state because it gets phosphorylated at S73 (reaction equation (2)), *k*_*Mp*73-_[*MITF*_*p*73_] is the rate of MITF entering this state from the S73 single phosphorylated state due to de-phosphorylation (reaction equation (2)), *k*_*Mp*73*a*+_[*MITF*] is the rate of MITF leaving this state because of auto-phosphorylation at S73 (reaction equation (3)), *k*_*Mp*409+_[*MITF*][*RSK*1*_p_*] is the rate of MITF leaving this state because of phosphorylation at S409 (reaction equation (4)), *k*_*Mp*409-_[*MITF*_*p*409_] is the rate of MITF entering this state from the S409 single phosphorylated MITF state because of de-phosphorylation (reaction equation (4)), *k_Mass_*[*MITF*][*PIAS*3] is the rate of MITF leaving this state because of MITF-PIAS3 association (reaction equation (5)) and *k_Mdiss_*[*PIAS*3_*MITF*] is the rate of MITF entering this state due to MITF-PIAS3 dissociation (reaction equation (5)). The last term *γ_MITF_*[*MITF*], represents the MITF degradation. The total amount of RSK1 is kept constant, while its phosphorylation state is dynamically determined by equations (12) and (13).

(12) $$\overset{•}{\left[RSK1\right]}=-k_{Rp+}\left[RSK1\right]\left[ERK_{p}\right]+k_{Rp-}\left[RSK1_{p}\right]$$

(13) $$\overset{•}{\left[RSK1_{p}\right]}=k_{Rp+}\left[RSK1\right]\left[ERK_{p}\right]-k_{Rp-}\left[RSK1_{p}\right]$$

The dynamics of S73 phosphorylated MITF (*MITF_p73_*) is given in equation (14)

(14) $$\begin{array}{c} \overset{•}{\left[MITF_{p73}\right]}=+k_{Mp73E}\left[ERK_{p}\right]\left[MITF\right]+k_{Mp73a+}\left[MITF\right]\\ -k_{Mp73-}\left[MITF_{p73}\right]\\ -k_{Mp409+}\left[RSK1_{p}\right]\left[MITF_{p73}\right]+k_{Mp409-}\left[MITF_{pp}\right]\\ -k_{Mp73ass}\left[MITF_{p73}\right]\left[PIAS3\right]\\ +k_{Mp73diss}\left[PIAS3\_MITF_{p73}\right]\\ -\gamma_{MITFp73}R\left[MITF_{p73}\right] \end{array}$$

Where *γ*_*MITFp*73 _is the degradation rate that applies to the ubiquitinated ratio *R *of the amount of *MITF*_*p*73_. In the dynamics of S409 phosphorylated MITF,

(15) $$\begin{array}{c} \overset{•}{\left[MITF_{p409}\right]}=-k_{Mp73E+}\left[ERK_{p}\right]\left[MITF_{p409}\right]-k_{Mp73a}-\left[MITF_{p409}\right]\\ +k_{Mp73-}\left[MITF_{pp}\right]\\ +k_{Mp409+}\left[RSK1_{p}\right]\left[MITF\right]-k_{Mp409-}\left[MITF_{p409}\right]\\ -k_{Mp409ass}\left[MITF_{p409}\right]\left[PIAS3\right]\\ +k_{Mp409diss}\left[PIAS3\_MITF_{p409}\right]\\ -\gamma_{MITFp409}\left[MITF_{p409}\right] \end{array}$$

simple linear degradation is applied. When MITF is phosphorylated on both sites, the sum of the two degradation rates applies:

(16) $$\begin{array}{c} \overset{•}{\left[MITF_{pp}\right]}=+k_{Mp73E}\left[ERK_{p}\right]\left[MITF_{p409}\right]+k_{Mp73a}+\left[MITF_{p409}\right]\\ -k_{Mp73-}\left[MITF_{pp}\right]\\ +k_{Mp409+}\left[RSK1_{p}\right]\left[MITF_{p73}\right]-k_{Mp409-}\left[MITF_{pp}\right]\\ -k_{Mppass}\left[MITF_{pp}\right]\left[PIAS3\right]+k_{Mppdiss}\left[PIAS3\_MITF_{pp}\right]\\ -\gamma_{MITFp73}R\left[MITF_{pp}\right]-\gamma_{MITFp409}\left[MITF_{pp}\right] \end{array}$$

Further, these four phosphorylation states of MITF bind PIAS3, each with their own association and dissociation rate constants, yielding differential equation (17) for PIAS3, (18) for the MITF-PIAS3-complex, (19) for the S73 phosphorylated MITF-PIAS3-complex, (20) for the S409 phosphorylated MITF-PIAS3-complex and (21) for the double phosphorylated MITF-PIAS3-complex.

(17) $$\begin{array}{c} \overset{•}{\left[PIAS3\right]}=+p_{PIAS3}\\ -k_{Mass}\left[PIAS3\right]\left[MITF\right]+k_{Mdiss}\left[PIAS3\_MITF\right]\\ -k_{Mp73ass}\left[PIAS3\right]\left[MITF_{p73}\right]\\ +k_{Mp73diss}\left[PIAS3\_MITF_{p73}\right]\\ -k_{Mp409ass}\left[PIAS3\right]\left[MITF_{p409}\right]\\ +k_{Mp409diss}\left[PIAS3\_MITF_{p409}\right]\\ -k_{Mppass}\left[PIAS3\right]\left[MITF_{pp}\right]+k_{Mppdiss}\left[PIAS3\_MITF_{pp}\right]\\ -k_{Spass}\left[PIAS3\right]\left[STAT3_{p}\right]+k_{Spdiss}\left[PIAS3\_STAT3_{p}\right]\\ -\gamma_{PIAS3}\left[PIAS3\right] \end{array}$$

PIAS3 has a linear degradation.

(18) $$\begin{array}{c} \overset{•}{\left[PIAS3\_MITF\right]}=-k_{Mp73E+}\left[ERK_{p}\right]\left[PIAS3\_MITF\right]\\ +k_{Mp73-}\left[PIAS3\_MITF_{p}{}_{73}\right]\\ -k_{Mp73a+}\left[PIAS3\_MITF\right]\\ -k_{Mp409+}\left[RSK1_{p}\right]\left[PIAS3\_MITF\right]\\ +k_{Mp409-}\left[PIAS3\_MITF_{p409}\right]\\ +k_{Mass}\left[MITF\right]\left[PIAS3\right]\\ -k_{Mdiss}\left[PIAS3\_MITF\right]\\ -\frac{\gamma_{MITF}+\gamma_{PIAS3}}{10}\left[PIAS3\_MIFTF\right] \end{array}$$

(19) $$\begin{array}{c} \overset{•}{\left[PIAS3\_MITF_{p73}\right]}=+k_{Mp73E+}\left[ERK_{p}\right]\left[PIAS3\_MITF\right]\\ -k_{Mp73-}\left[PIAS3\_MITF_{p}{}_{73}\right]\\ +k_{Mp73a+}\left[PIAS3\_MITF\right]\\ -k_{Mp409+}\left[RSK1_{p}\right]\left[PIAS3\_MITF_{p73}\right]\\ +k_{Mp409-}\left[PIAS3\_MITF_{pp}\right]\\ +k_{Mp73ass}\left[MITF_{p73}\right]\left[PIAS3\right]\\ -k_{Mp73diss}\left[PIAS3\_MITF_{p73}\right]\\ -\frac{\gamma_{MITFp73}+\gamma_{PIAS3}}{10}R\left[PIAS3\_MITF_{p73}\right] \end{array}$$

(20) $$\begin{array}{c} \overset{•}{\left[PIAS3\_MITF_{p409}\right]}=-k_{Mp73E+}\left[ERK_{p}\right]\left[PIAS3\_MITF_{p409}\right]\\ +k_{Mp73-}\left[PIAS3\_MITF_{pp}\right]\\ -k_{Mp73a+}\left[PIAS3\_MITF_{p409}\right]\\ +k_{Mp409+}\left[RSK1_{p}\right]\left[PIAS3\_MITF\right]\\ -k_{Mp409-}\left[PIAS3\_MITF_{p409}\right]\\ +k_{Mp409ass}\left[MITF_{p409}\right]\left[PIAS3\right]\\ -k_{Mp409diss}\left[PIAS3\_MITF_{p409}\right]\\ -\frac{\gamma_{MITFp409}+\gamma_{PIAS3}}{10}\left[PIAS3\_MITF_{p409}\right] \end{array}$$

(21) $$\begin{array}{c} \overset{•}{\left[PIAS3\_MITF_{pp}\right]}=+k_{Mp73E+}\left[ERK_{p}\right]\left[PIAS3\_MITF_{p409}\right]\\ -k_{Mp73-}\left[PIAS3\_MITF_{pp}\right]\\ +k_{Mp73a+}\left[PIAS3\_MITF_{p409}\right]\\ +k_{Mp409+}\left[RSK1_{p}\right]\left[PIAS3\_MITF_{p73}\right]\\ -k_{Mp409-}\left[PIAS3\_MITF_{pp}\right]\\ +k_{Mppass}\left[MITF_{pp}\right]\left[PIAS3\right]\\ -k_{Mppdiss}\left[PIAS3\_MITF_{pp}\right]\\ -\frac{\gamma_{MITFp73}R+\gamma_{PIAS3}}{10}\left[PIAS3\_MITF_{pp}\right]\\ -\frac{\gamma_{MITFp409}+\gamma_{PIAS3}}{10}\left[PIAS3\_MITF_{pp}\right] \end{array}$$

STAT3 is binding PIAS3 while phosphorylated. The dynamics of the PIAS3-STAT3-complex is given in equation (22) and un-phosphorylated and phosphorylated STAT3 in equation (23) and (24) respectively.

(22) $$\begin{array}{c} \overset{•}{\left[PIAS3\_STAT3_{p}\right]}=+k_{Spass}\left[STAT3_{p}\right]\left[PIAS3\right]\\ -k_{Spdiss}\left[PIAS3\_STAT3_{p}\right]\\ -\frac{\gamma_{STAT3p}+\gamma_{PIAS3}}{10}\left[PIAS3\_STAT3_{p}\right] \end{array}$$

To represent higher stability by proteins in complexes, the degradation rate of the complex is set to 20% of the mean of the degradation rates assigned to the constituting proteins in un-bound form.

(23) $$\begin{array}{c} \overset{•}{\left[STAT3\right]}=+p_{STAT3}\\ -k_{Sp+}\left[JAK_{p}\right]\left[STAT3\right]+k_{Sp-}\left[STAT3_{p}\right]\\ -\gamma_{STAT3}\left[STAT3\right] \end{array}$$

(24) $$\begin{array}{c} \overset{•}{\left[STAT3_{p}\right]}=+k_{Sp+}\left[JAK_{p}\right]\left[STAT3\right]-k_{Sp-}\left[STAT3_{p}\right]\\ -k_{Spass}\left[STAT3_{p}\right]\left[PIAS3\right]+k_{Spdiss}\left[PIAS3\_STAT3_{p}\right]\\ -\gamma_{STAT3}\left[STAT3_{p}\right] \end{array}$$

S73 phosphorylated MITF get ubiquitin tagged for degradation by phosphorylated ERK. Introducing another modification site would cause another doubling of the number of state variables representing MITF. To reduce complexity, we chose to represent the ubiquitination with one differential equation (25), describing the ratio *R *of the total amount of S73 phosphorylated MITF that also is ubiquitinated.

(25) $$\begin{array}{ccc} \overset{∙}{R}= & \left(1-R\right)\left[ERK_{p}\right]k_{u} & \left(i\right)\\ & -\left(R-\frac{1-{\gamma_{MITF}}_{p73}a}{\frac{1}{R}-{\gamma_{MITF}}_{p73}a}\right)\frac{1}{a} & \left(ii\right),\\ & -\left(R-RA\right)\frac{1}{a} & \left(iii\right) \end{array}$$

Where *A *is defined as

(26) $$A=\frac{\left[MITF_{p73}\right]+\left[MITF_{pp}\right]}{\left[MITF_{p73}\right]+\left[MITF_{pp}\right]+\left(p_{MITF}-\left[MITF_{p409}\right]\gamma_{MITFp409}\right)a}$$

The three parts of equation (25) correspond to the three processes altering the ratio R: (i) The ubiquitination of un-ubiquitinated MITF, (ii) the ubiquitination dependent degradation of ubiquitinated MITF and (iii) the production of new un-ubiquitinated MITF. In (i), the ubiquitination rate constant *k_u_*, denotes the rate of change of R and thus have the unit au^-1 ^min^-1^. To derive part (ii), let *R*' be the ratio *R *after one minute of ubiquitin mediated degradation. Then the rate of change due to this effect is -(*R - R*') per minute, where

(27) $$R^{\prime }=\frac{RB-RB\gamma_{MITFp73}a}{B-B\gamma_{MITFp73}a}=\frac{1-\gamma_{MITFp73}a}{\frac{1}{R}-\gamma_{MITFp73}a}$$

and *B *= [*MITF*_*p*73_] + [*MITF_pp_*]. The term *a *= 1 and has the unit minute and does not affect the calculation, but is needed for the correct unit representation. Part (iii) is derived in a similar way: Let *R*'' be the ratio *R *after one minute of MITF production. Then the rate of change due to this effect is -(*R - R*'') per minute, where

(28) $$R\prime \prime =\frac{R\left(\left[MITF_{p73}\right]+\left[MITF_{pp}\right]\right)}{\left[MITF_{p73}\right]+\left[MITF_{pp}\right]+\left(p_{MITF}-\left[MITF_{p409}\right]\gamma_{MITFp409}\right)a}.$$

### MITF activity

The readouts of the model are the amounts of the different phosphorylation and complex states of the proteins involved. To be able to view this model in the light of available experimental results, these levels have to be interpreted in a way comparable to the lab-generated data. The transcriptional activity of MITF and STAT3 is measured either as luciferase activity of a transfected reporter gene or as mRNA-levels of downstream genes.

We thus devised a function *f*([*MITF*],[*MITF*_*p*73_],[*MITF*_*p*409_],[*MITF*_*p*p_]) to map the amounts of the different phosphorylation states of MITF to a downstream transcriptional activity. The production of any gene product can be modelled by a function *f*(*S*_1_,*S*_2_,....,*S_N_*), where *S_j _*is the concentration of the *N *species affecting the transcription and translation of the particular gene product. This function can be described at various levels of precision from the basic principles of chemical kinetics, via Michaelis-Menten enzymatic kinetics to low order polynomials that function as an ansatz for the actual molecular mechanisms. The function that maps from the amounts of the MITF phosphorylation states to production rate of downstream genes will probably differ between target genes on a higher resolution level. However, for the resolution level of the current effort, one mapping function is sufficient. We have approximated the MITF activity by a sum of first order polynomials, which in effect is a weighted sum:

(29) $$\begin{array}{ccc} f= & \;A_{0}+A_{M}\left[MITF\right]+A_{M73}\left[MITF_{p73}\right] & \\ & \;+A_{M409}\left[MITF_{p409}\right]+A_{Mpp}\left[MITF_{pp}\right] \end{array}$$

Values for the transcriptional activity of different MITF mutants are given in [14]. From these we have calculated the factors to be *A_M _*= -0.11, *A*_*M*73 _= 0.44, *A*_*M*409 _= 0.11 and *A*_*Mpp *_= 0.56. This simple representation of MITF transcriptional activity is able to take negative values, which does not make sense biologically; therefore, *A*_0 _is set to the relative high arbitrary value 10, which is sufficiently high to avoid negative MITF activity in this study.

### Parameterizing the model

While this model contains a large number of parameters, we can make at least a reasonable order of magnitude estimate for most of them from empirical biological data. In the experiments used for model development and parameter fitting, neither implicit nor explicit absolute protein amounts have been provided. The cells are either transfected, activated or both, and thus the input protein amounts and perturbations will by this fact be qualitative. The results are presented as qualitative protein or complex amounts (Western blot) or transcriptional activity for MITF and PIAS3 (measured by luciferase assays or PCR-measurements of target genes mRNA levels). These results are quantified relative to a control, but not in absolute terms.

An estimate for the degradation rates of PIAS3 and STAT3 is calculated directly from the protein stability index (PSI) as presented in [25] (*γ*_*STAT*3 _= 0.002 and *γ*_*PIAS*3 _= 0.008). The PSI derived degradation rate for MITF is 0.008, but MITF is known to be relatively stable in un-phosphorylated form while ERK mediated phosphorylation on S73 also tags MITF for proteasome mediated degradation [13,14]. To match degradation time series data [13,14] MITF was assigned a low degradation rate in the un-phosphorylated state, *γ*_*MITF *_= 0.0012, and relatively high degradation rate when phosphorylated on S409 (regardless of phosphorylation status on S73), *γ*_*MITFp*73 _= 0.01. The proportion of MITF phosphorylated on S73 (regardless of phosphorylation status on S409) is degraded only when ubiquitinated (and then with a rather high degradation rate, *γ*_*MITFp*73 _= 0.02). The ubiquitination rate constant is adjusted to *k*_*u *_= 0.0001 to yield degradation in accordance with data [13,14]. When the proteins are in complex, they are thought to be more stable than as free molecules. As an approximation the degradation rates of all complexes were set to 20% of the average of the degradation rate of the two components.

The production rates for MITF, PIAS3 and STAT3 are complex functions of the concentration of a large number of substances, of which none are involved in this model. Therefore these production rates are represented here by constants whose values are relative to the corresponding degradation rates determined by the steady state levels of each of the three proteins. It is important for the proper function of this module that these three proteins are present at approximately equimolar levels. The values are therefore set to *p_MITF _*= 1, *p_PLAS3 _*= 1.262 and *p*_*STAT*3 _= 0.211 to meet this criterion. This yields a steady state amount of approximately 100 (au). The level of phosphorylated ERK in the resting cell is set to *ERK*_p _= 10 and is elevated to approximately 1000 whenever a MAPK signal is simulated. The total amount of RSK1 is set to 500 of which the phosphorylation is determined dynamically (equation (12) and (13)). The phosphorylation speed for RSK1 is found in [14], and the phosphorylation and de-phosphorylation rate constants calculated to *k*_*Rp*+ _= 0.0004 and *k*_*Rp*- _= 0.04, respectively. The Western blot analysis of MITF presented in [13] and [14] has one band interpreted as un-phosphorylated MITF and one band interpreted as phosphorylated MITF. No interpretation is provided on which phosphorylation states this latter band may represent. By interpreting this band as the sum of all phosphorylated MITF, we have adjusted the phosphorylation rate constants of MITF to make the model behaviour fit the experimental data. The rate constants are set to *k*_*Mp*73*E*+ _= 0.00015, *k*_*Mp73*- _= 0.03, *k*_*Mp*73*a*+ _= 0.025, *k*_*Mp*409+ _= 0.0001 and *k*_*Mp*409- _= 0.04. With no direct measurements of the STAT3 phosphorylation rate, we assigned the same values to the STAT3 phosphorylation/de-phosphorylation rate constants as we did for RSK1: *k*_*Sp*+ _= 0.0002 and *k*_*Sp*- _= 0.04. The constant representing phosphorylated JAK is set to 10 in the un-activated state and approximately 1000 in the activated state.

With no direct measurements of the MITF-PIAS3 and STAT3-PIAS3 association and dissociation rate constants, they were inferred from qualitative results and assumptions. We anticipated that this process is faster than the phosphorylation process, which means that both association and dissociation rate constants should be relatively high. MITF has lower affinity to PIAS3 when phosphorylated at S409 and higher affinity when phosphorylated at S73, compared to un-phosphorylated MITF [20]. The double phosphorylated MITF exhibits an intermediate affinity comparable to that of un-phosphorylated MITF [20]. When the MITF-PIAS3 association/dissociation rate constants were adjusted so that the model emulated the results from [20], they were assigned the following values: *k*_*Miss *_= 0.01, *k*_*Mdiss *_= 1, *k*_*Mp*73*ass *_= 0.03, *k*_*Mp*73*diss *_= 0.5, *k*_*Mp*409*ass *_= 0.0001, *k*_*Mp*409*diss *_= 1, *k*_*Mppass *_= 0.01 and *k*_*Mppdss *_= 1. The STAT3-PIAS3 association/dissociation rate constants were adjusted in the same way to data from [22] and are assigned the values *k*_*Spass *_= 0.005 and *k*_*Spass *_= 0.2.

### Formalizing the experiments

We extracted a set of representative experiments from four relevant publications [14,19,20,22] (Table 2). The numbering in the table provided a unique ID for each experiment, and was used throughout the article. The perturbations of the cells that were reported in the published experiments, like transfection of mutated genes or receptor activation, were assigned digital counterparts in the model through alterations of the input values, starting values, or some of the parameters. In the following, these details, as well as the criteria used for the sensitivity analysis, are given for each of the experiments.

Experiment # 1 is a simulation of the experiment presented in [14] Figure 5B. Here, the authors use kinase assays to investigate the temporal development of ERK and RSK1 kinase activity after stimulation of melanoma cells. The stimulation was simulated by elevation of the amount of phosphorylated ERK to 1000 from the default value of 10. The success criterion used in the sensitivity analysis was that the phosphorylated RSK1 level should reach 90% of its maximum between 3 and 10 minutes after activation.

Experiment #2 simulates the pre-stimulation distribution among the MITF phosphorylation states in the experiments presented in [14], Figure 1 and 2. To emulate the growing cell culture the level of phosphorylated ERK was elevated from the default value of 10 to the intermediate level of 80. Steady state values were found for the state variables and the total amount of phosphorylated and un-phosphorylated MITF was compared. The success criterion used in the sensitivity analysis was that both categories should be between 25% and 75% of the total amount.

Experiment #3 is also based on [14], Figure 1 and 2. Here, the phosphorylation state of MITF after 30 minutes of activation is considered. The cell stimulation is simulated by elevation of the levels of phosphorylated ERK and JAK from the default value of 10 to 1000. The success criterion used in the sensitivity analysis was that more than 80% of the MITF is phosphorylated after 30 minutes.

Experiment #4 is also based on [14], Figure 1 and 2. Here, the degradation profile is considered. The cell stimulation is simulated by elevation of the levels of phosphorylated ERK and JAK from the default value of 10 to 950. The success criterion used in the sensitivity analysis is that less than 50% of the MITF is degraded after 1 h and at least two thirds are degraded after 5 h.

Experiment #5 is a simulation of the experiment presented in [19], Figure 6. Here, the authors have transfected MITF, a MITF reporter gene, and different amounts of PIAS3 into NIH 3T3 fibroblasts (endogenously not expressing MITF). To mimic MITF transfection, the MITF production rate is increased from 1 to 18. To mimic the four different amounts of PIAS3 transfection, the PIAS3 production rate was increased from 0.211 to 0.5, 1, 2 and 4. The model was simulated for 2880 minutes (2 days) for each perturbation and the resulting MITF activity was compared for the four PIAS3 levels. The success criterion used in the sensitivity analysis was that MITF activity should be monotonically decreasing with increasing PIAS3 and that the MITF activity at the highest PIAS3 amount should be less than the half that of the lowest PIAS3 amount.

In experiments #6 and #7, we have mimicked the experiments presented in [20], Figure 2. Here the authors have investigated the association between MITF and PIAS3 in BL6-B16 cells before and after 10 and 30 minutes of activation with c-kit ligand. In experiment #6 only GFP-PIAS3 was transfected, while in experiment #7, the cells were transfected with both MITF and PIAS3. In experiment #6, activation is performed by increase of the levels of phosphorylated ERK and JAK from 10 to 500 and 50, respectively. In experiment #7, transfection was simulated by elevation of the MITF production rate from 1 to 10 and the PIAS3 production rate from 1.262 to 10 and simulated for 2880 minutes (2 days). Thereafter, activation was simulated by increase of the amount of phosphorylated ERK and JAK from 10 to 1200 and 250, respectively. The success criterion used for the sensitivity analysis in both experiments was that the total amount of MITF-PIAS3-complex should be higher after 10 minutes of stimulation compared to before stimulation and that the amount of complex should be lower after 30 minutes of stimulation than after 10 minutes.

Experiment #8 simulated the experiment from [20], Figure 3A, left. The authors co-transfected NIH 3T3 cells using MITF, with a constitutively active RSK1 plasmid and with a reporter gene to read MITF activity. To simulate transfection of MITF, the MITF production rate was elevated from 1 to 7. The amount of RSK1 is not dynamically determined in the model, and thus the transfection of this product is approximated with an elevation of the static amount from 500 to 5000. The manipulated model was simulated for 2880 minutes (2 days), and the MITF activities with and without RSK1 were compared. The success criteria used in the sensitivity analysis was that the two cases should not differ more than twofold.

Experiments #9 to #12 represent simulations of the experiment presented in [20] Figure 3B. Here, the authors transfected NIH 3T3 cells with MITF or the MITF-S409A mutant, with constitutively active RSK1 and with a reporter gene to monitor MITF transcriptional activity. To simulate the transfection of MITF, the MITF production rate was elevated from 1 to 7. To simulate the transfection of PIAS3, the PIAS3 production rate was elevated from 1.262 to 4. The amount of RSK1 is not dynamically determined in the model, and thus the transfection of this product is approximated with an elevation of the static amount from 500 to 5000. The activation level was elevated from 10 to 20 to emulate the activity in growing cells. After simulation for 2880 minutes (2 days), the MITF activities, with and without PIAS3 were compared for each of the four experiments. For each experiment the manipulations were as follows: without RSK1, with wild-type MITF (experiment #9), with RSK1 and wild-type MITF (experiment #10), without RSK1, with mutant MITF (experiment #10) and with RSK1, with mutant MITF (experiment #11). The success criterion used in the sensitivity analysis was for each experiment: Experiment #9: MITF activity in the case with PIAS3 is less than 42% and greater than 2% of that without PIAS3. Experiment #10: MITF activity in the case of PIAS3 is less than 84% and greater than 44% of that without PIAS3. Experiment #11: MITF activity in the case of PIAS3 is less than 55% and greater than 15% of that without PIAS3. Experiment #12: MITF activity in the case of PIAS3 is less than 50% and greater than 10% of that without PIAS3.

Experiment #13 to #16 are simulations of the experiment presented in [20] Figure 6. In this experiment the authors investigate the inhibition of transcriptional activity of S/D mutants of MITF by PIAS3. This is achieved by transfection of wild-type MITF (experiment #13), MITF-S73D (experiment #14), MITF-S409D (experiment # 15) or MITF-S73/409D (experiment #16) and PIAS3 and a reporter gene into NIH 3T3 cells. We simulated the MITF transfection by elevation of the MITF production rate from 1 to 1.5, and the PIAS3 transfection was simulated by elevation of the PIAS3 production rate from 1.262 to 4.5. Here we have also set all MITF levels to zero before starting the simulation, since NIH 3T3 fibroblasts do not express endogenous MITF. To simulate MITF-S73D mutation we have set the MITF S73 de-phosphorylation rate constant to zero while the MITF S73 auto-phosphorylation rate constant is elevated from 0.025 to 5. To simulate MITF-S409D mutation, the RSK1 phosphorylation rate constant is elevated form 0.0004 to 0.04, the RSK1 de-phosphorylation rate constant is decreased from 0.04 to 0.004, the MITF S409 phosphorylation rate constant is elevated from 0.0001 to 0.01 and the MITF S409 de-phosphorylation rate constant is set to zero. The model was simulated for 2880 minutes (2 days) and the MITF transcriptional activity with PIAS3 was compared with the case without PIAS3 for wild-type MITF, and for each mutation form. The success criterion used for the sensitivity analysis was for each experiment: Experiment #13: MITF activity in the case of PIAS3 is less than 70% and greater than 30% of that without PIAS3. Experiment #14: MITF activity in the case of PIAS3 is less than 50% and greater than 10% of that without PIAS3. Experiment #15: MITF activity in the case of PIAS3 is less than 115% and greater than 75% of that without PIAS3. Experiment #16: MITF activity in the case of PIAS3 is less than 85% and greater than 45% of that without PIAS3.

Experiments #17 and #18 are simulations of the experiment presented in [22] Figure 3A. Here, the authors investigate PIAS3-STAT3 association in response to stimulation of NIH 3T3 cells transfected with STAT3, PIAS3 and MITF or the MITF S409A-mutant. The transfection is simulated by elevation of the MITF production rate from 1 to 10, the PIAS3 production rate from 1.262 to 10 and the STAT3 production rate from 0.211 to 10. The MITF S409A mutation was simulated by setting the MITF S409 phosphorylation rate constant to zero. The activation was simulated by elevation of the amount of phosphorylated ERK and JAK from 10 to 1000. In experiment #17, wild-type MITF, PIAS3 and STAT3 were all transfected and simulated for 2880 minutes (2 days), thereafter simulated for 15 more minutes with and without stimulation. The amount of PIAS3-STAT3-complex was compared. The success criterion used in the sensitivity analysis was that the amount of PIAS3-STAT3-complex should be at least 25% higher with stimulation compared to the case without stimulation. In experiment #18, S409A MITF, PIAS3 and STAT3 were transfected and simulated for 2880 minutes (2 days), and thereafter simulated for 15 more minutes with or without stimulation. The amount of PIAS3-STAT3-complex was compared with the stimulated case in experiment #17. The success criterion used in the sensitivity analysis was that the amount of PIAS3-STAT3-complex in the stimulated case in experiment #17 should be at least 25% higher than the amount of PIAS3-STAT3-complex in any of the two cases in #18.

Experiments #19 to #24 are simulations of the experiment presented in [22] Figure 3B. Here, the authors have investigated STAT3 transcriptional activity as a response to activation, transfection of PIAS3, and transfection of various amounts of wild-type and S409A mutated MITF in NIH T3T cells. The activation was simulated by elevation of the amounts of phosphorylated ERK and JAK from 10 to 1000. The transfections of PIAS3, STAT3 and the two different MITF amounts were simulated by elevation of the PIAS3 production rate from 1.262 to 7, the STAT3 production rate from 0.262 to 5, and the MITF production rate from 1 to 10 or 50, respectively. The S409A mutation was simulated by setting the MITF phosphorylation rate constant to zero. The model was run for 2880 minutes to simulate the incubation and then for 360 minutes to simulate the activation. The background luciferase activity of STAT3 was determined by elevation of the STAT3 production rate and running a model simulation for 3240 (2880+360) minutes. The amount of phosphorylated STAT3 in the end was interpreted as an estimate for the luciferase activity of STAT3 without activation and without PIAS3 or MITF. For experiments #19 to #24, the amount of phosphorylated STAT3 was compared to this background level. In experiment #19, the cells were transfected with STAT3 and activated. The success criterion used in the sensitivity analysis was the level of phosphorylated STAT3 between 10 and 20 times the background level. In experiment #20, the cells were transfected with STAT3 and PIAS3 and activated. The success criterion used in the sensitivity analysis was the level of phosphorylated STAT3 between 2.67 and 5.33 times the background level. In experiment #21 the cells were transfected with STAT3, PIAS3 and the smaller amount of MITF and activated. The success criterion used in the sensitivity analysis was the level of phosphorylated STAT3 between 2.67 and 5.33 times the background level. In experiment #22 the cells were transfected with STAT3, PIAS3 and the larger amount of MITF and activated. The success criterion used in the sensitivity analysis was the level of phosphorylated STAT3 between 6.67 and 13.33 times the background level. In experiment #23 the cells were transfected with STAT3, PIAS3 and the smaller amount of S409A mutated MITF and activated. The success criterion used in the sensitivity analysis was the level of phosphorylated STAT3 between 5.67 and 11.33 times the background level. In experiment #24 the cells were transfected with STAT3, PIAS3 and the larger amount of S409A mutated MITF. The success criterion used in the sensitivity analysis was the level of phosphorylated STAT3 between 10 and 20 times the background level.

Experiment #25 simulated the experiment presented in [22] Figure 4B. Here, the authors have investigated MITF transcriptional activity in response to transfection of PIAS3, STAT3-Y705F mutant and a constitutively active STAT3 mutant (STAT3-C) in NIH 3T3 cells. The transfections were simulated by elevation of the production rate of MITF from 1 to 2, PIAS3 from 1.262 to 2 and STAT3 from 0.211 to 20. The NIH 3T3 cells were simulated by decreasing the starting values of all MITF and PIAS3 states to 1 and in simulations where MITF or PIAS3 were not transfected; their production rate were set to zero. The STAT3-Y705F mutant was simulated by setting the STAT3 phosphorylation rate constant to zero. STAT3-C was simulated by increase the STAT3 phosphorylation rate constant from 0.0002 to 0.02 and setting the de-phosphorylation rate constant to zero. Six different simulations were performed and the MITF transcriptional activity was compared: (i) Cells transfected with STAT3-Y705F, (ii) cells transfected with STAT3-C, (iii) cells transfected with MITF and STAT3-Y705F, (iv) cells transfected with MITF and STAT3-C, (v) cells transfected with MITF, PIAS3 and STAT3-Y705F and (vi) cells transfected with MITF, PIAS3 and STAT3-Y705F. The success criterion used in the sensitivity analysis was that the MITF transcriptional activity from simulation (i) should be between 50% and 150% of the MITF activity form (ii) and the MITF activity from (iii) should be between 80% and 120% of the MITF activity from (iv) and the MITF activity from (v) should be between 12.5% and 100% of the MITF activity from (vi) and the MITF activity from (i) should be less than the MITF activity from (iii) and the MITF activity from (iii) should be greater than 200% of the MITF activity from (v).

Experiment #26 simulates the experiment presented in [22] Figure 6D and 6E. Here, the authors use mRNA levels of MITF and STAT3 target genes to investigate MITF and STAT3 transcriptional activity after stimulation of mast cells derived from wild type or mutant mice. The MITF protein from the mutated mice lacks its ability to bind PIAS3. The activation is simulated by elevation of the amounts of phosphorylated ERK and JAK from 10 to 1000. The mutation is simulated by setting all four MITF-PIAS3 association rate constants to zero. The success criterion used in the sensitivity analysis was for cells from wild type mice: MITF activity after 30 minutes should be more than twice the MITF activity without stimulation, STAT3 activity at 4 hours should be more than twice the STAT3 activity without stimulation and for cells from mutated mice: MITF activity at 30 minutes and at 4 hours should be less than twice the MITF activity without stimulation and STAT3 activity at 30 minutes and 4 hours should be less than twice the STAT3 activity without stimulation.

Experiments #27 and #28 are simulations of the experiments presented in [22] Figure 7C and 7D. In experiment #27 the authors have investigated the STAT3 transcriptional activity in response to transfection of wild type MITF and a MITF mutant emulating MITF phosphorylated at S409. In experiment #28 the authors have investigated MITF transcriptional activity in response to transfection of constitutively active STAT3 (STAT3-C). The transfections were simulated by elevation of the STAT3 production rate from 0.211 to 5 and the MITF production rate from 1 to 5. The MITF mutant was simulated by setting the MITF-S409 phosphorylation rate constant to 5 and the MITF-S409 de-phosphorylation rate constant to zero. The STAT3-C mutant was simulated by increasing the STAT3 phosphorylation rate by 25 times and setting the de-phosphorylation rate to zero. The model was simulated for 2880 minutes without and with each of the three transfections. The success criterion used in the sensitivity analysis was for experiment #27 that the STAT3 activity had increased when the cells were transfected with MITF or the MITF mutant compared to without transfection and that the increase was higher when cells were transfected with wild type MITF compared to mutated MITF. The success criterion used in the sensitivity analysis for experiment #28 was MITF activity increased by at least 10%.

### Sensitivity analysis

The ability of the model to mimic the experiments described above was tested for values of the core parameters in the multidimensional neighbourhood of the default values. Each parameter was sampled from a distribution being uniform on the logarithmic scale, with a sampling area reaching from one half to double the default value. For each set of values of the core parameters, the experiments were simulated and the result (success or failure) was recorded for each experiment. This procedure was repeated 10^6 ^times resulting in 10^6 ^parameter sets, each with a corresponding result vector recording success or failure for each experiment.

In order to study the sensitivity of each experiment *i*'s success rate to variation in a parameter *j*, we sorted all parameter sets according to parameter *j *and divided them into 100 bins of 10000 parameter sets each. We used *f_i _*, the o verall success rate of experiment *i *(see Table 2, column 6) and $f_{i,j}^{k}$, the success rate in bin *k *to computed the sensitivity measure $s_{i,j}=\overset{100}{\underset{k=1}{\sum }}|f_{i,j}^{k}-f_{i}|$. In order to remove clearly insensitive parameters, we set to zero all *s_i, j _*that did not exceed the maximum value observed under 10000 permutations of the parameter values.

## Authors' contributions

JT, SWO and EH conceived the paper. JT designed the model, generated the parameter set, performed the programming and analysis of the model, and drafted the manuscript. TJL, ABG and SWO participated in model definition and revisions. TJL and EH contributed with knowledge on the biological scope, and with parameter estimation. ABG and JT performed the sensitivity analysis. All authors revised the manuscript. All authors read and approved the final manuscript.

## Supplementary Material

Additional file 1 **A zip-file containing temporal plots for all variables for all simulated experiments with default parameters**. The figures are in jpg-format. Click here for file

Additional file 2 **A zip-file containing an MATLAB implementation of the model and scripts used to carry out this study**. In addition to the MATLAB scripts, a readme file is provided with instructions on how to get the model running. Click here for file
