# Supplementary figures and images for: Towards a quantitative understanding of the MITF-PIAS3-STAT3 connection
Source: BMC Syst Biol. 2012 Feb 8;6:11. doi: 10.1186/1752-0509-6-11 (PMC3341200; doi:10.1186/1752-0509-6-11)

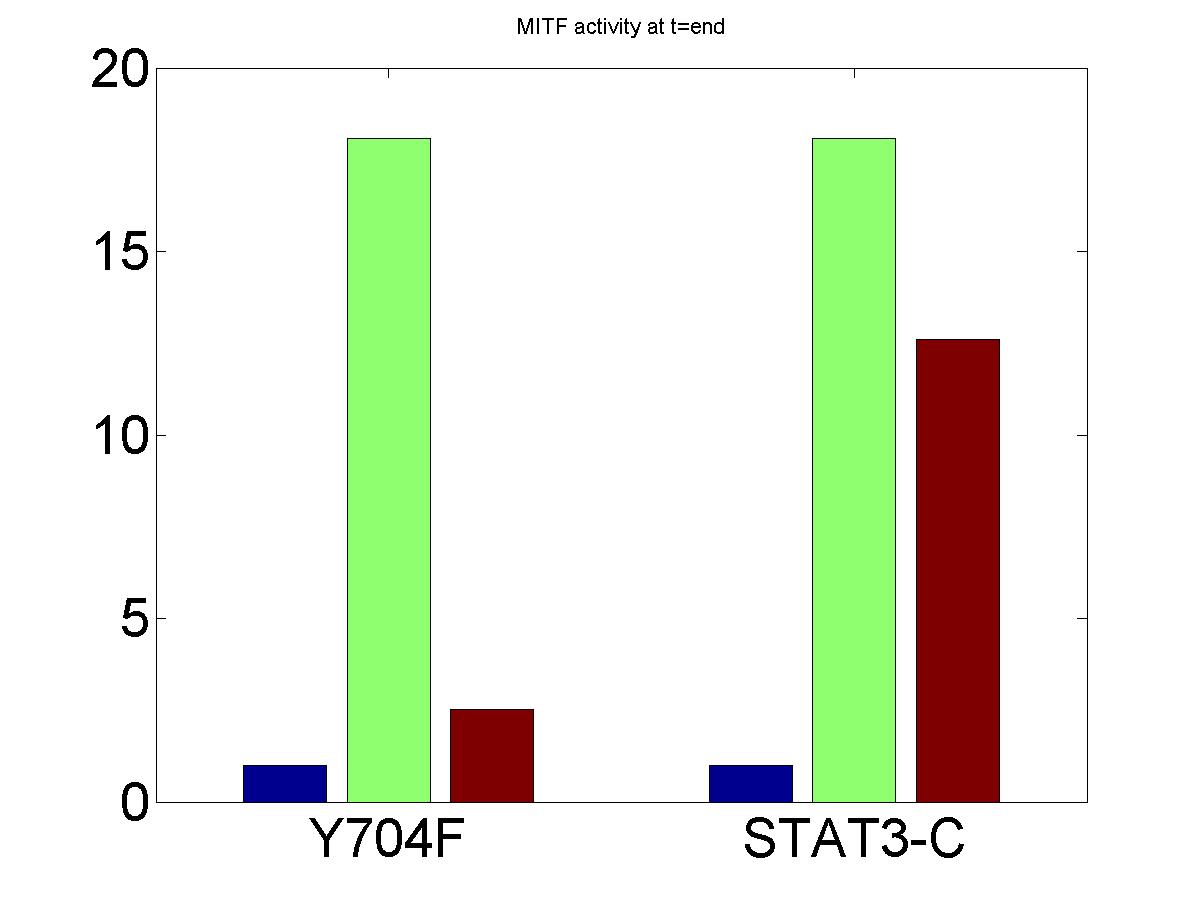

Supplement: Additional file 1 — A zip-file containing temporal plots for all variables for all simulated experiments with default parameters. The figures are in jpg-format. [file 1752-0509-6-11-S1.ZIP › simulationExperiment25b.jpg]

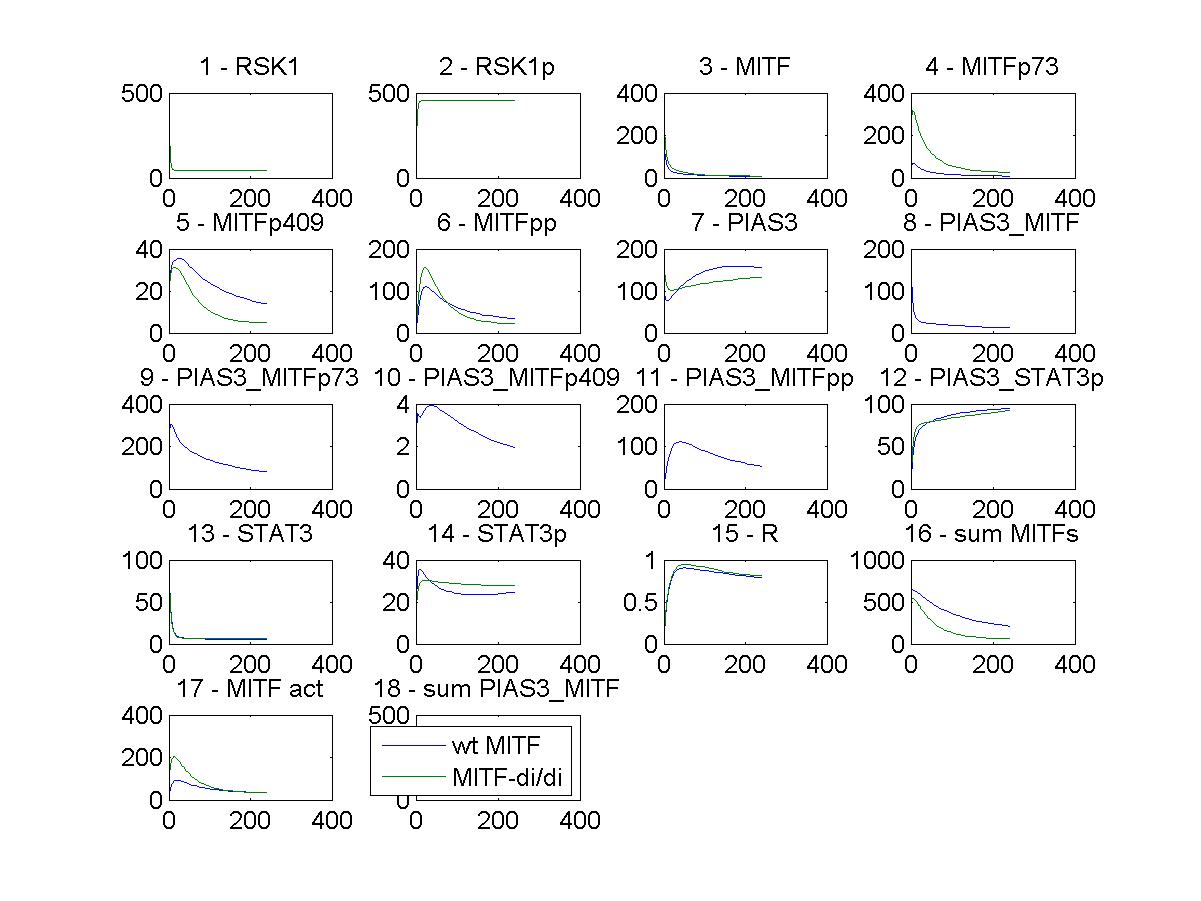

Supplement: Additional file 1 — A zip-file containing temporal plots for all variables for all simulated experiments with default parameters. The figures are in jpg-format. [file 1752-0509-6-11-S1.ZIP › simulationExperiment26(27).jpg]

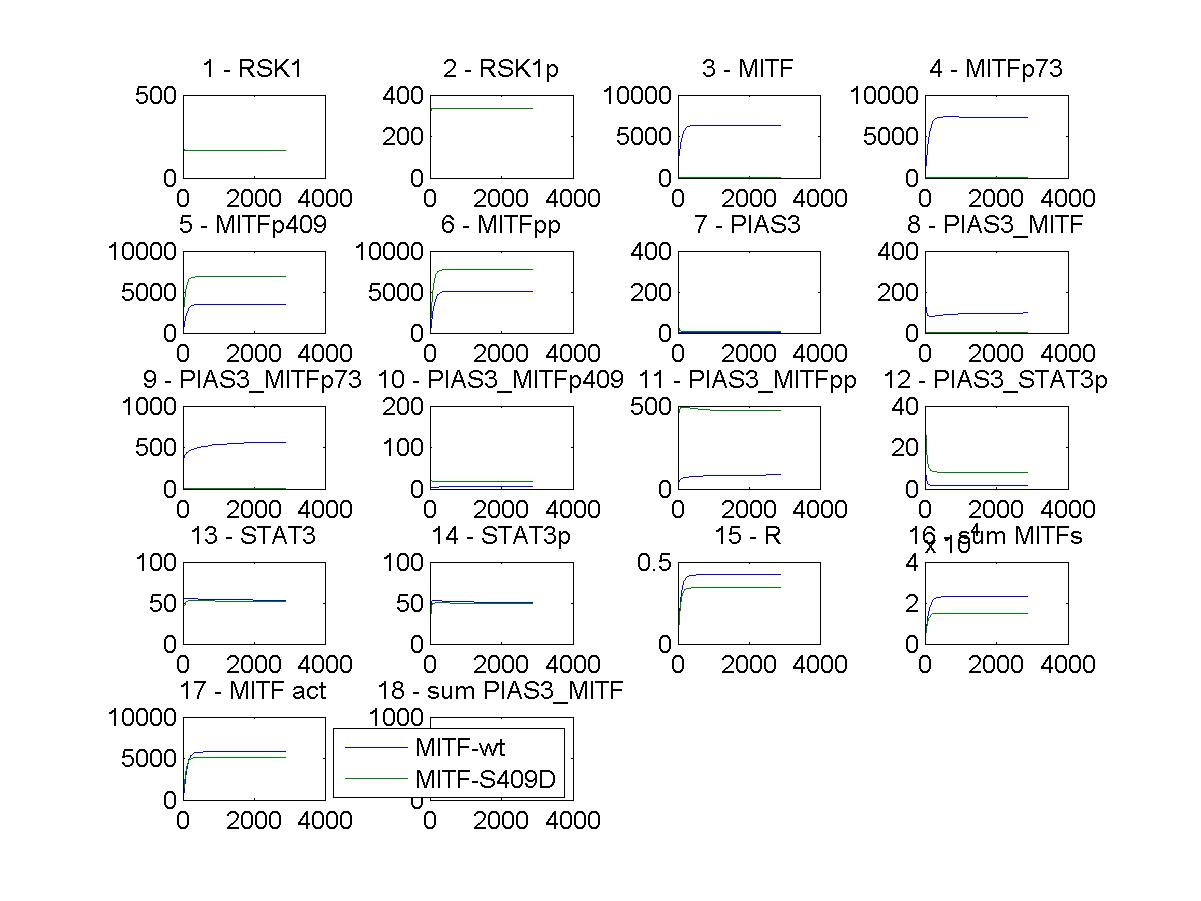

Supplement: Additional file 1 — A zip-file containing temporal plots for all variables for all simulated experiments with default parameters. The figures are in jpg-format. [file 1752-0509-6-11-S1.ZIP › simulationExperiment27(28).jpg]

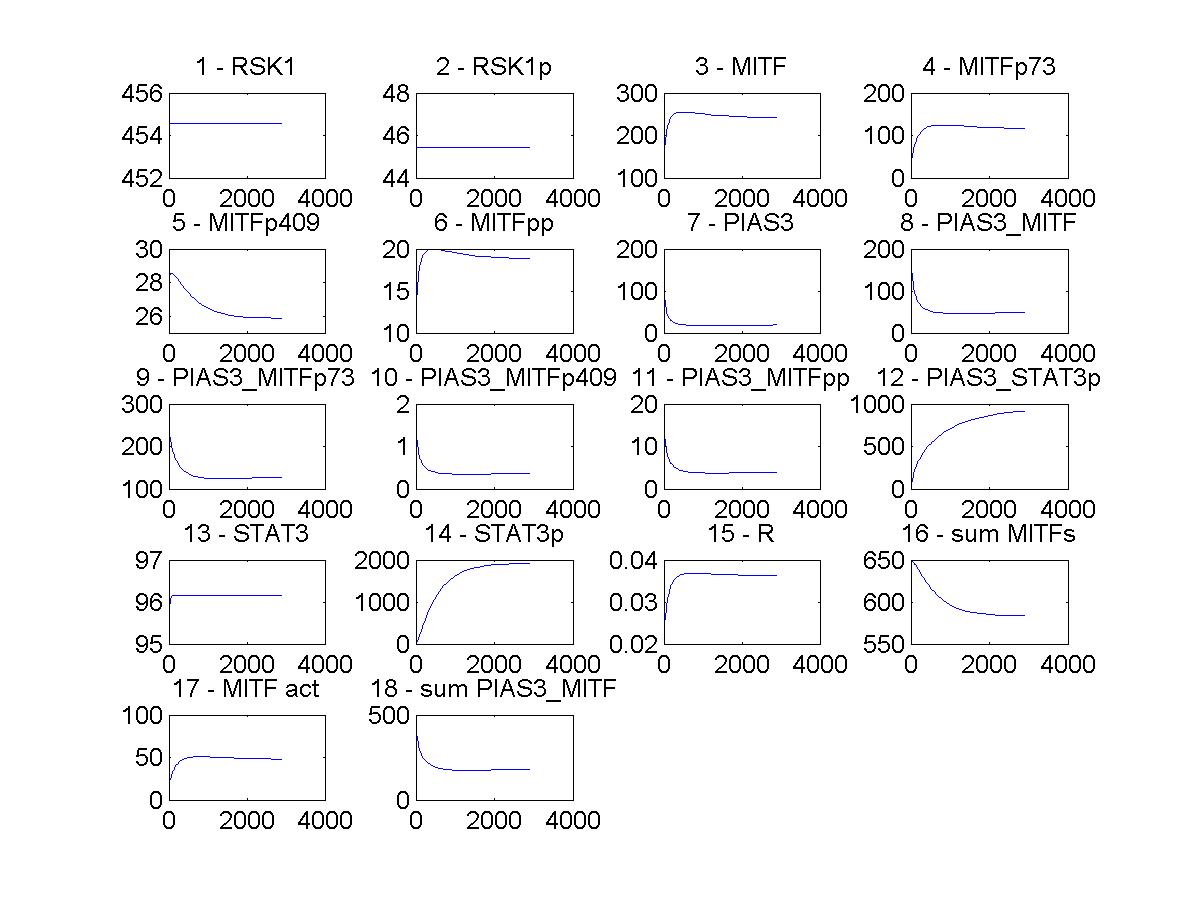

Supplement: Additional file 1 — A zip-file containing temporal plots for all variables for all simulated experiments with default parameters. The figures are in jpg-format. [file 1752-0509-6-11-S1.ZIP › simulationExperiment28(29).jpg]

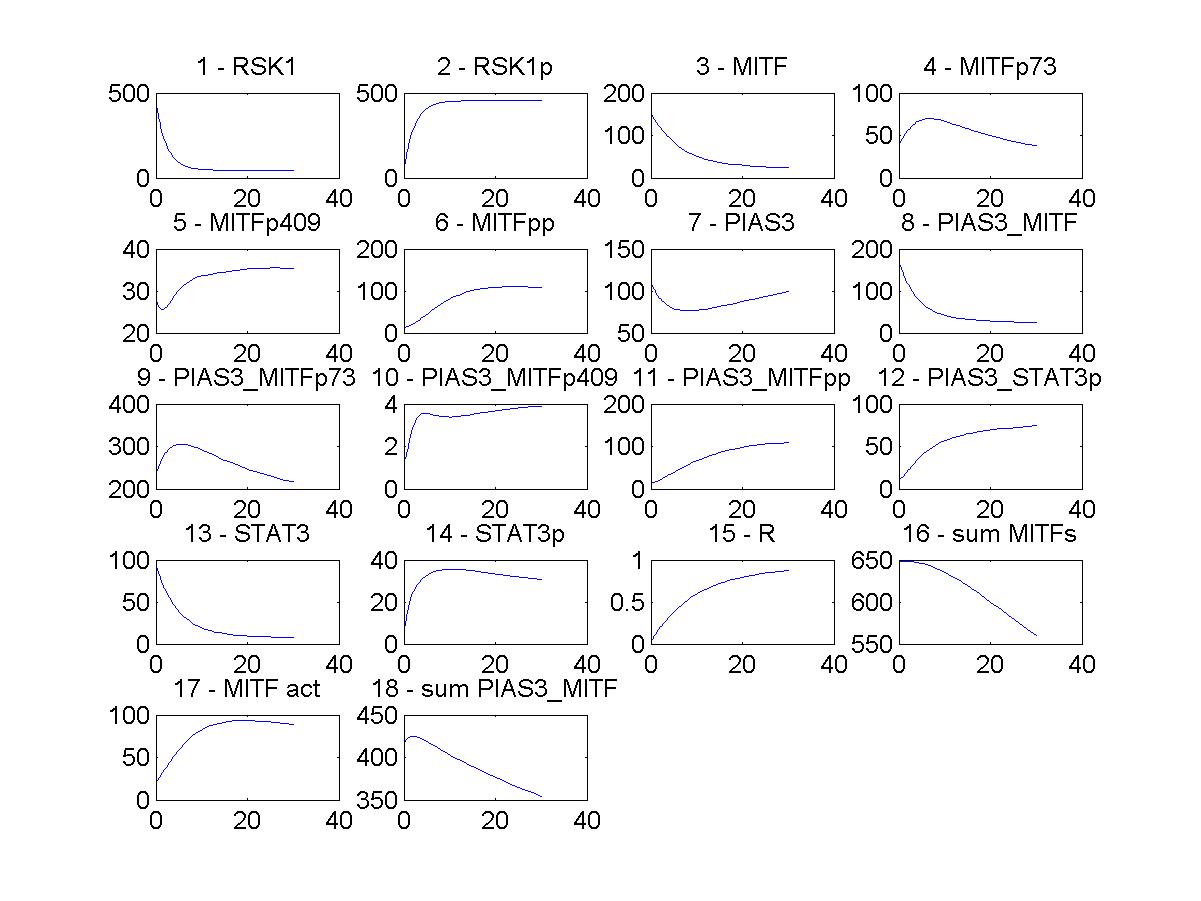

Supplement: Additional file 1 — A zip-file containing temporal plots for all variables for all simulated experiments with default parameters. The figures are in jpg-format. [file 1752-0509-6-11-S1.ZIP › simulationExperiment1.jpg]

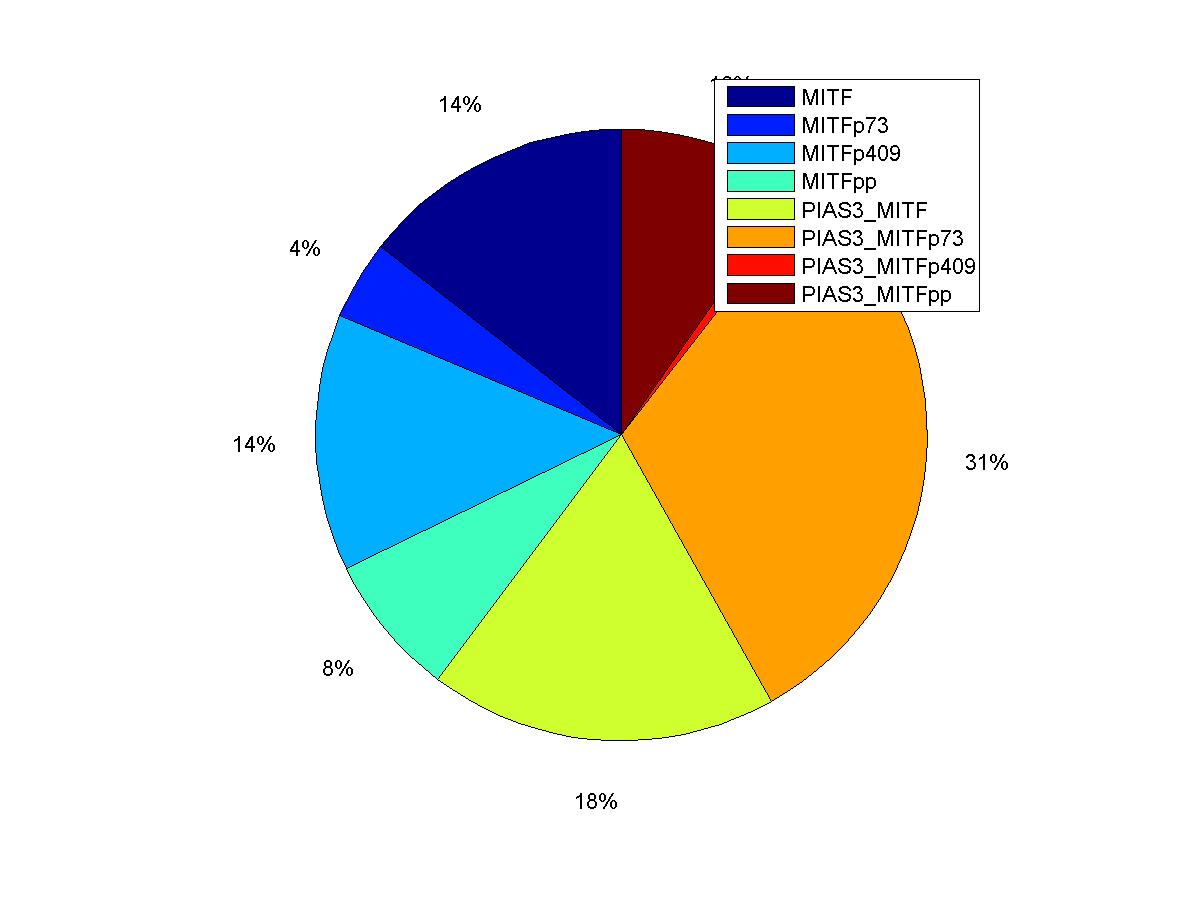

Supplement: Additional file 1 — A zip-file containing temporal plots for all variables for all simulated experiments with default parameters. The figures are in jpg-format. [file 1752-0509-6-11-S1.ZIP › simulationExperiment2.jpg]

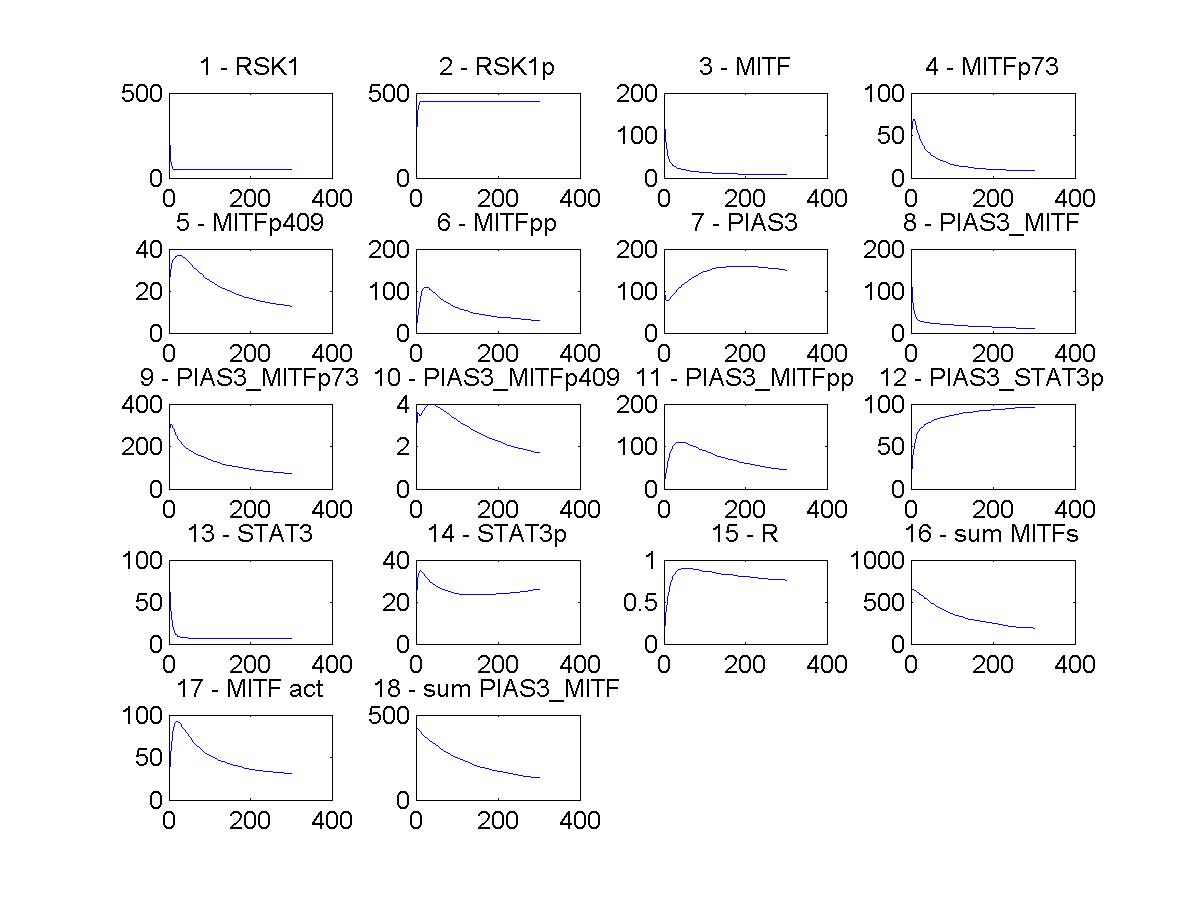

Supplement: Additional file 1 — A zip-file containing temporal plots for all variables for all simulated experiments with default parameters. The figures are in jpg-format. [file 1752-0509-6-11-S1.ZIP › simulationExperiment4.jpg]

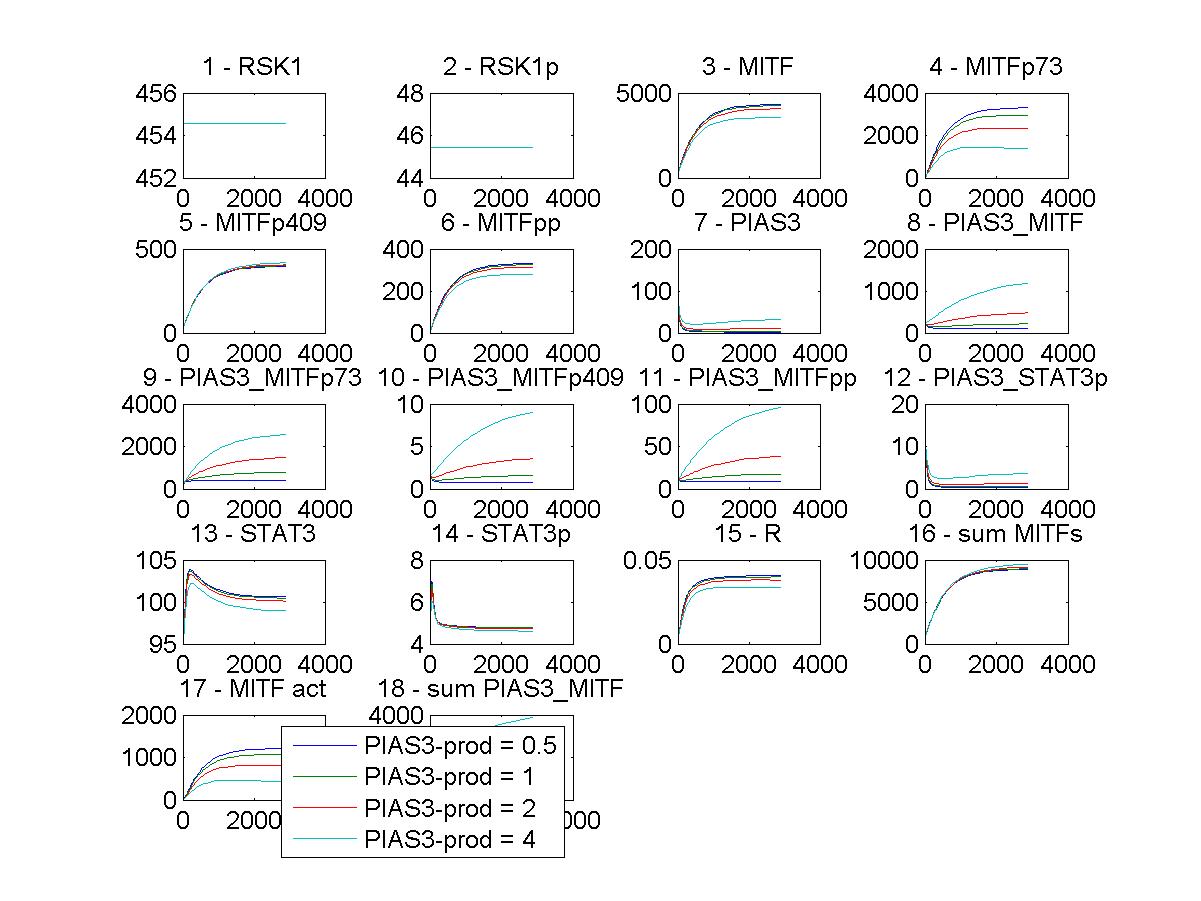

Supplement: Additional file 1 — A zip-file containing temporal plots for all variables for all simulated experiments with default parameters. The figures are in jpg-format. [file 1752-0509-6-11-S1.ZIP › simulationExperiment5.jpg]

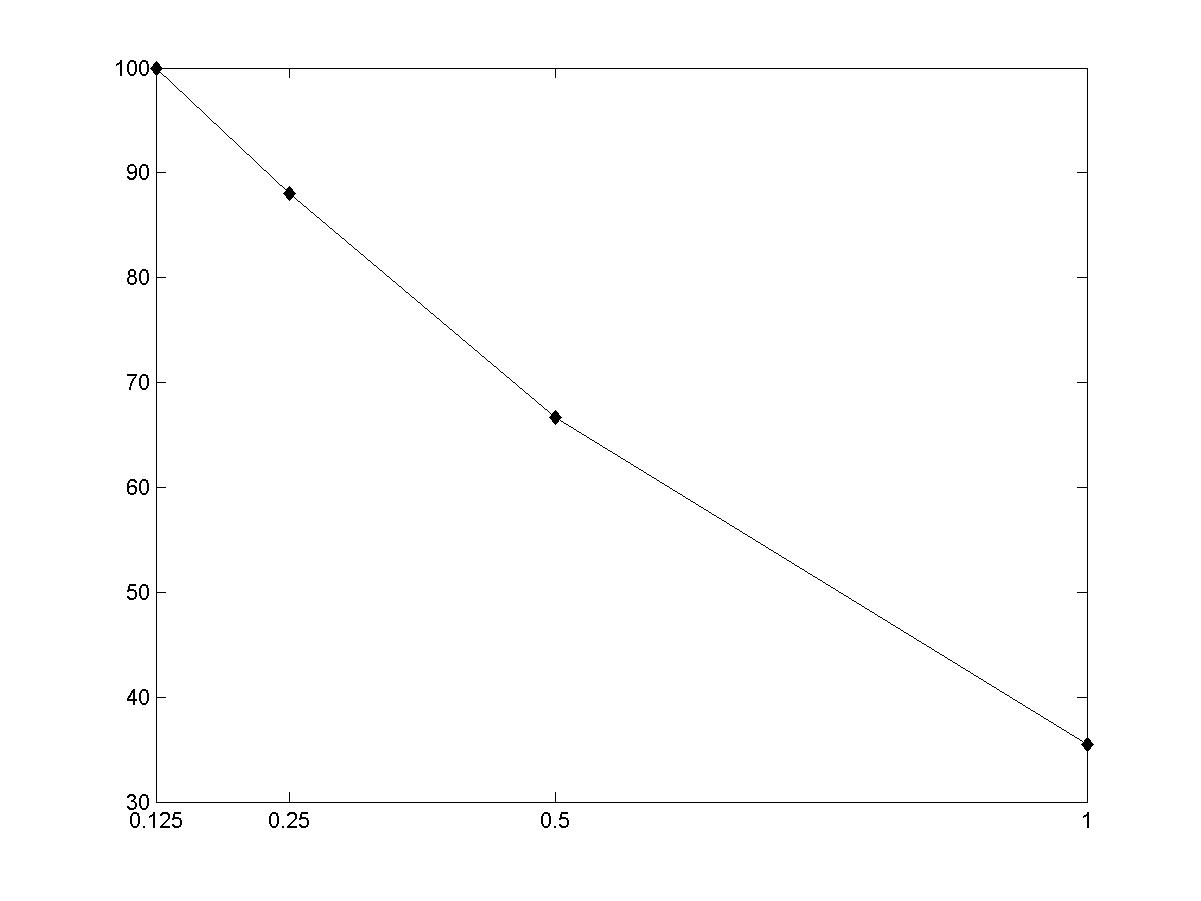

Supplement: Additional file 1 — A zip-file containing temporal plots for all variables for all simulated experiments with default parameters. The figures are in jpg-format. [file 1752-0509-6-11-S1.ZIP › simulationExperiment5b.jpg]

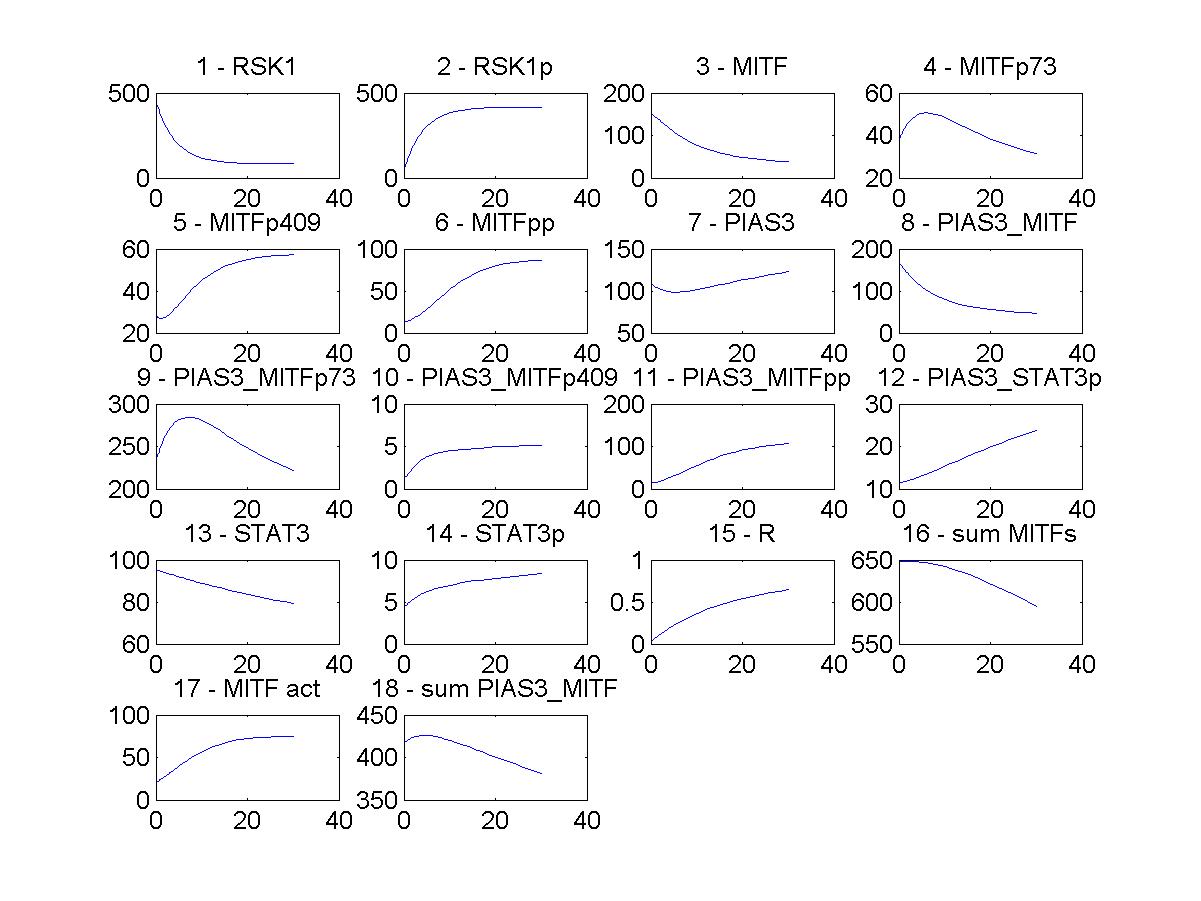

Supplement: Additional file 1 — A zip-file containing temporal plots for all variables for all simulated experiments with default parameters. The figures are in jpg-format. [file 1752-0509-6-11-S1.ZIP › simulationExperiment6.jpg]

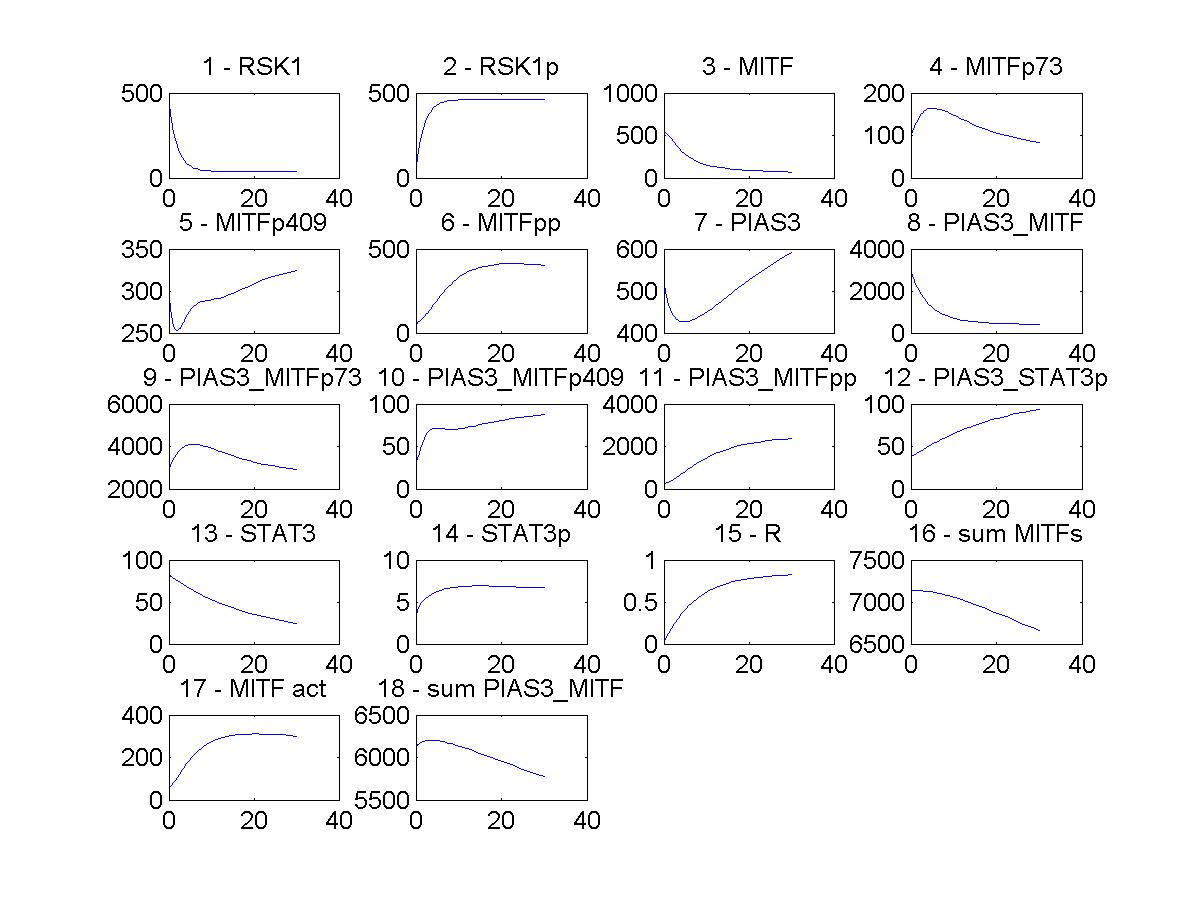

Supplement: Additional file 1 — A zip-file containing temporal plots for all variables for all simulated experiments with default parameters. The figures are in jpg-format. [file 1752-0509-6-11-S1.ZIP › simulationExperiment7.jpg]

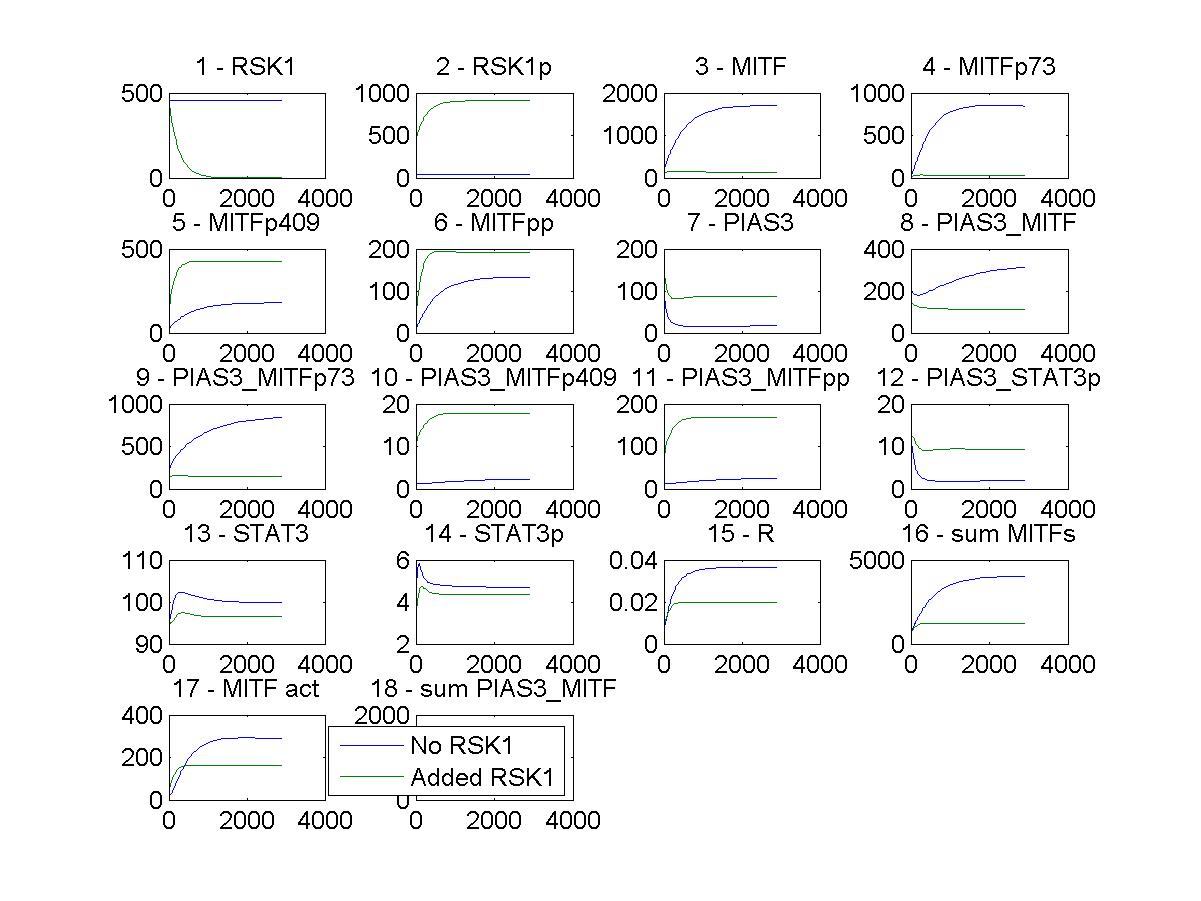

Supplement: Additional file 1 — A zip-file containing temporal plots for all variables for all simulated experiments with default parameters. The figures are in jpg-format. [file 1752-0509-6-11-S1.ZIP › simulationExperiment8.jpg]

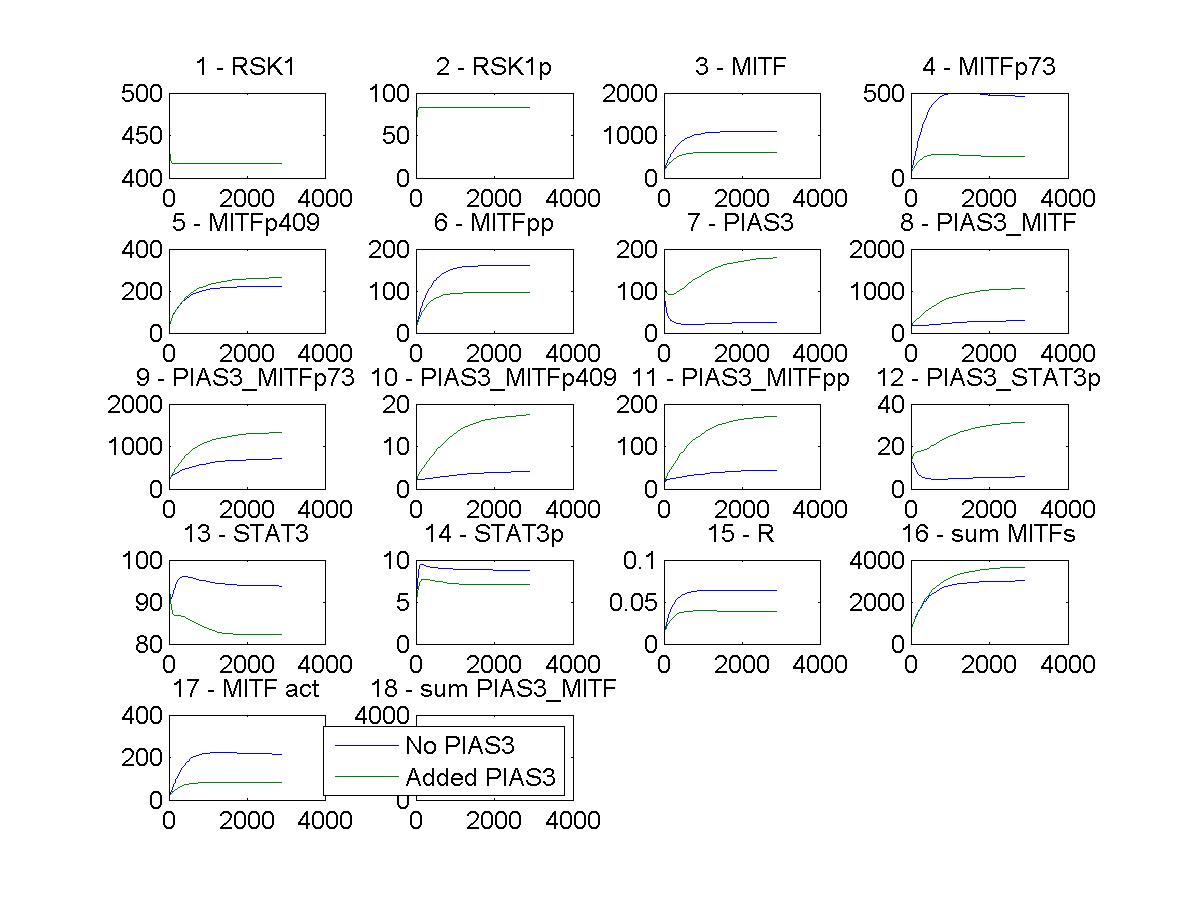

Supplement: Additional file 1 — A zip-file containing temporal plots for all variables for all simulated experiments with default parameters. The figures are in jpg-format. [file 1752-0509-6-11-S1.ZIP › simulationExperiment9.jpg]

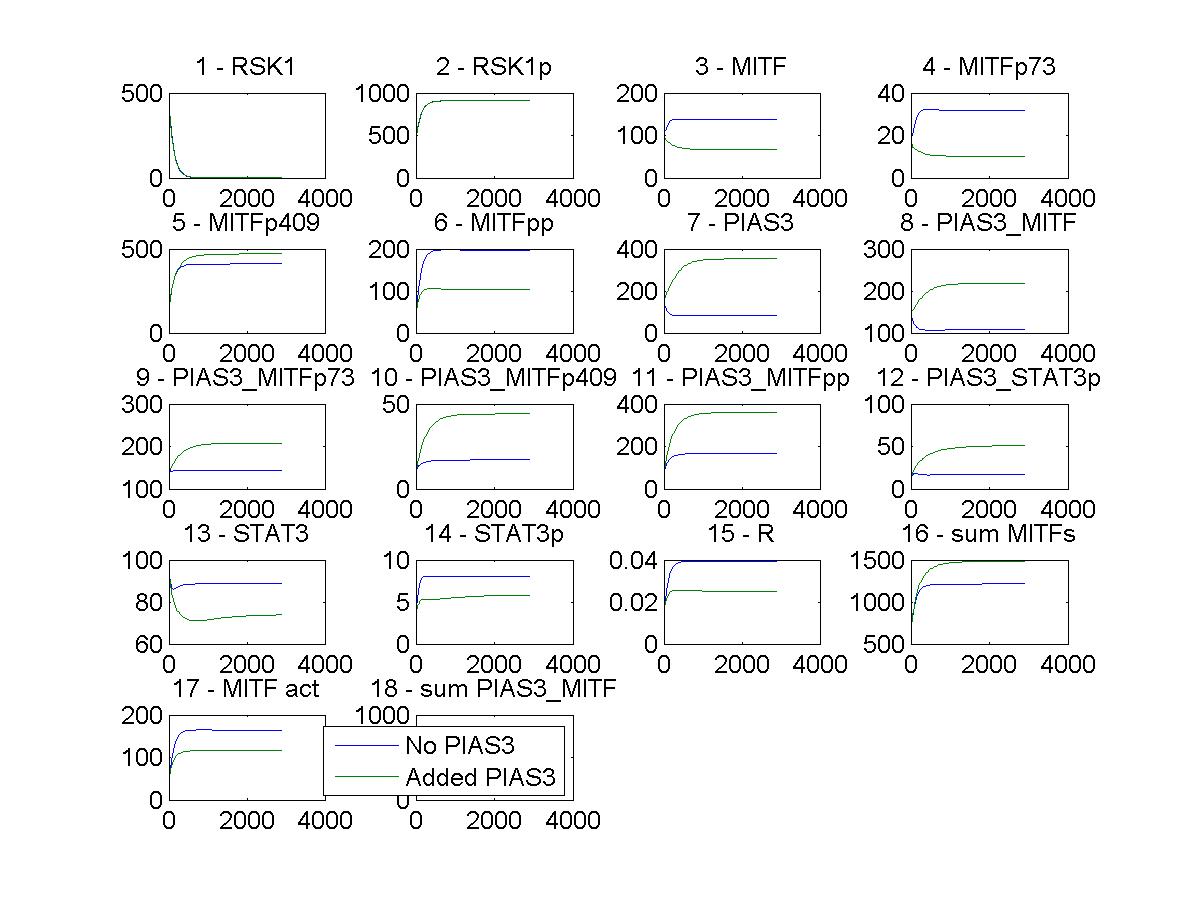

Supplement: Additional file 1 — A zip-file containing temporal plots for all variables for all simulated experiments with default parameters. The figures are in jpg-format. [file 1752-0509-6-11-S1.ZIP › simulationExperiment10.jpg]

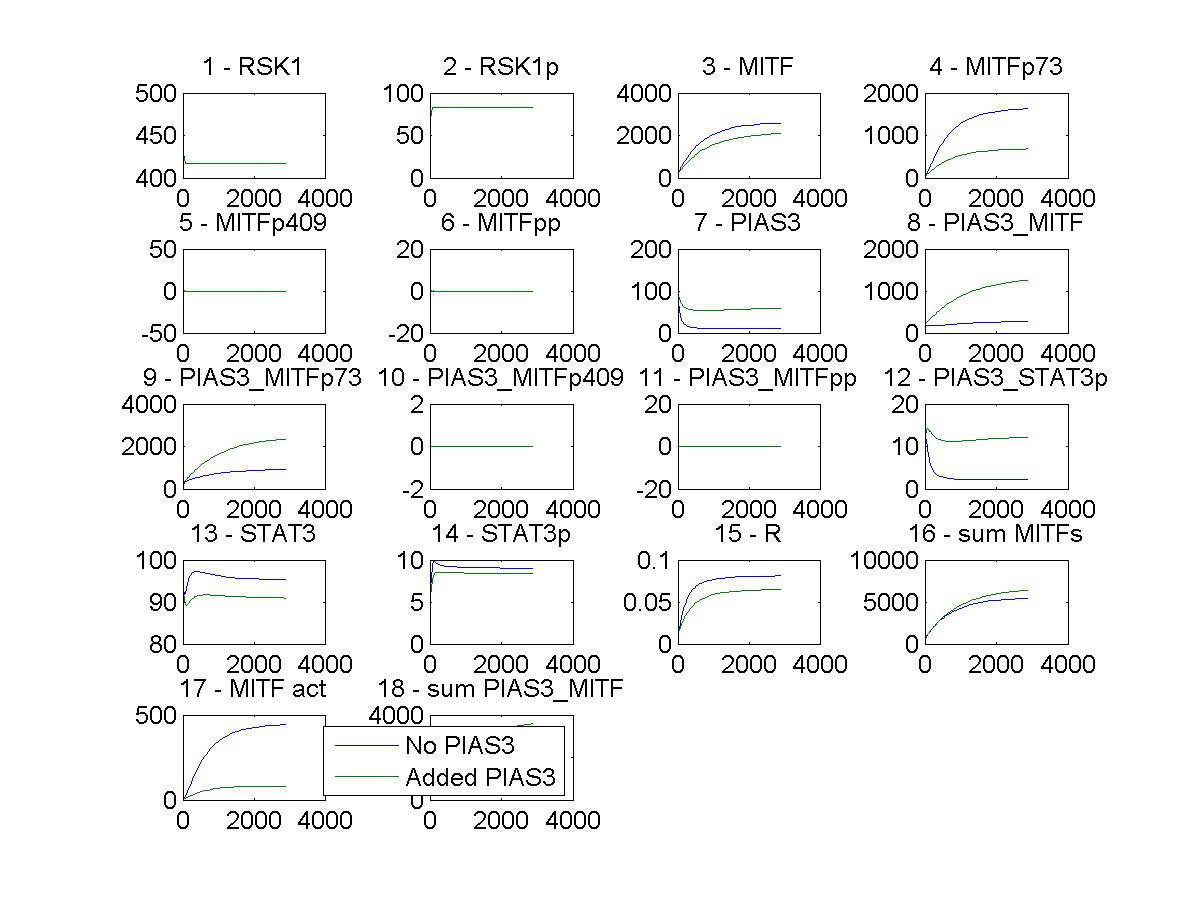

Supplement: Additional file 1 — A zip-file containing temporal plots for all variables for all simulated experiments with default parameters. The figures are in jpg-format. [file 1752-0509-6-11-S1.ZIP › simulationExperiment11.jpg]

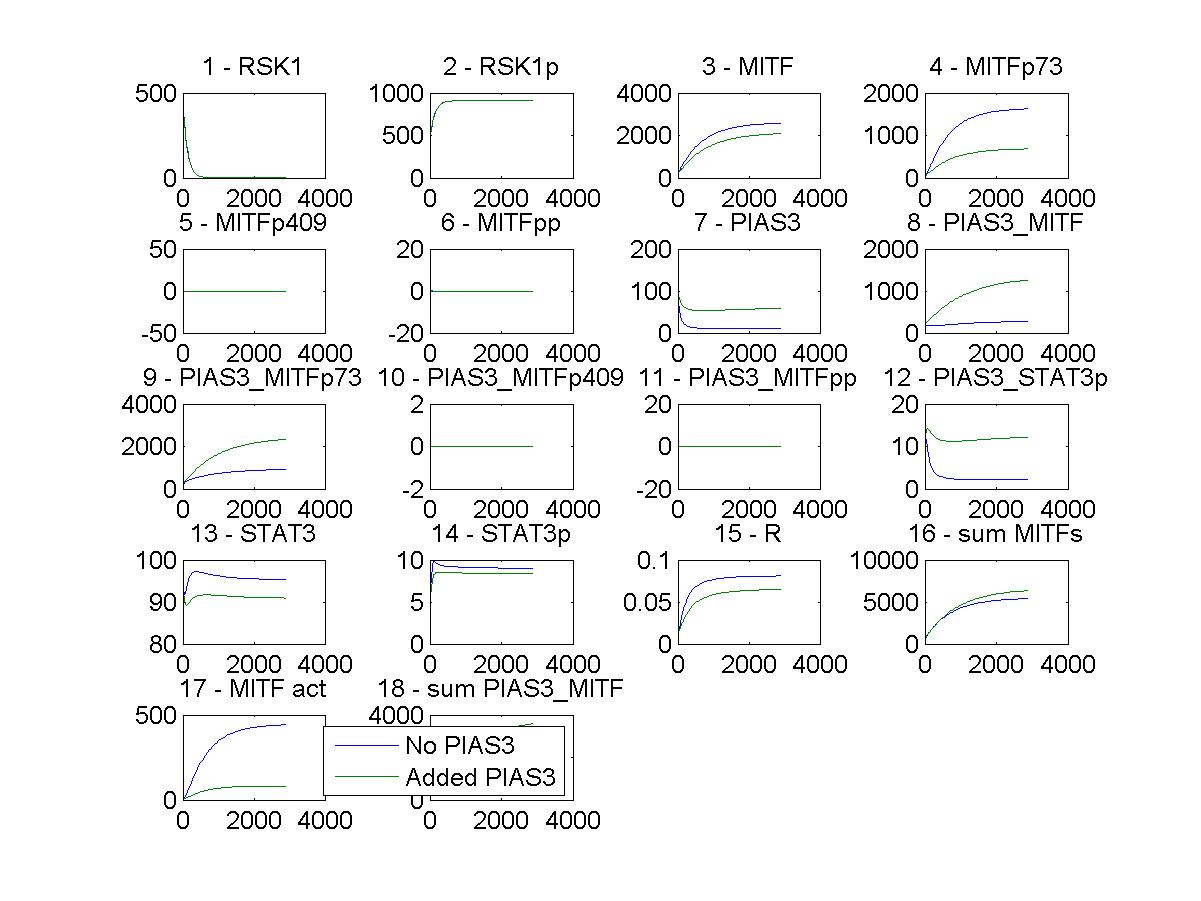

Supplement: Additional file 1 — A zip-file containing temporal plots for all variables for all simulated experiments with default parameters. The figures are in jpg-format. [file 1752-0509-6-11-S1.ZIP › simulationExperiment12.jpg]

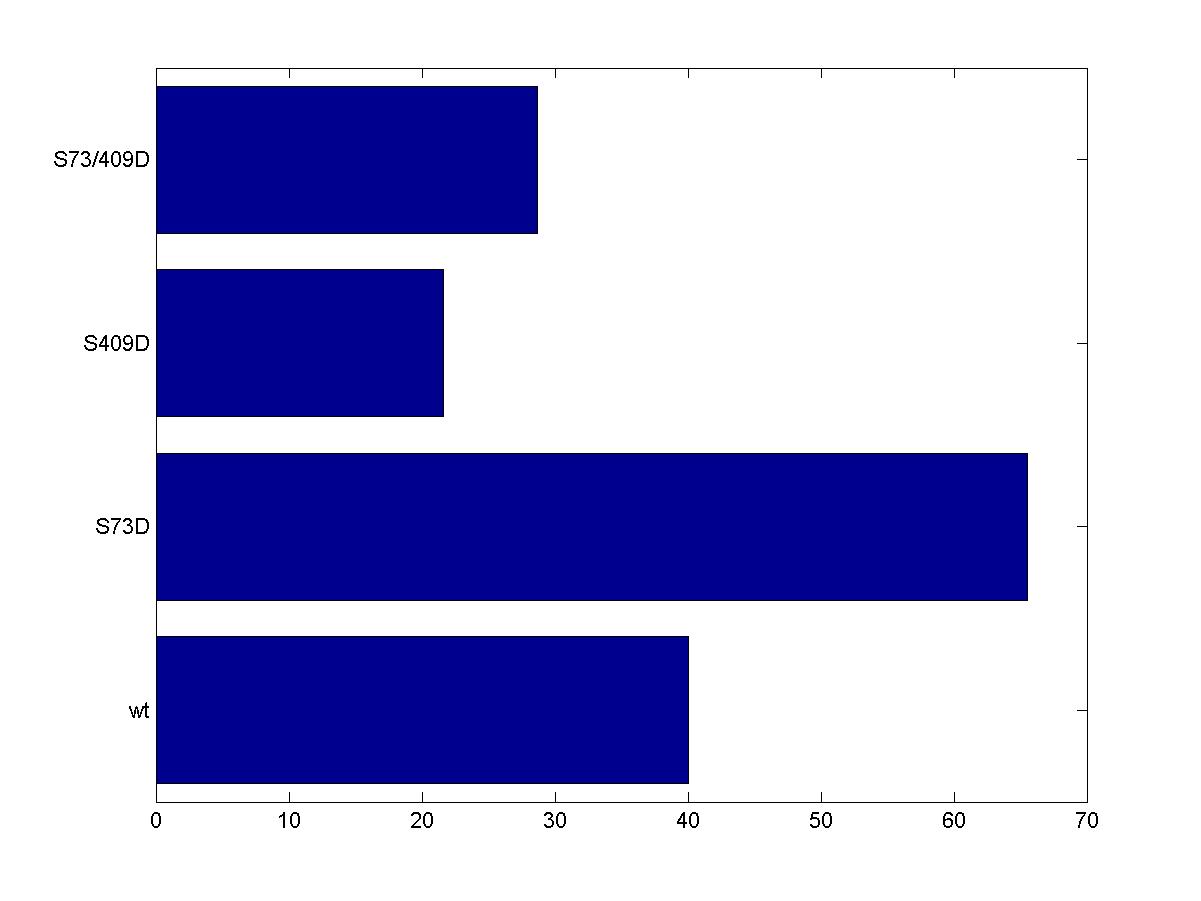

Supplement: Additional file 1 — A zip-file containing temporal plots for all variables for all simulated experiments with default parameters. The figures are in jpg-format. [file 1752-0509-6-11-S1.ZIP › simulationExperiment13-16.jpg]

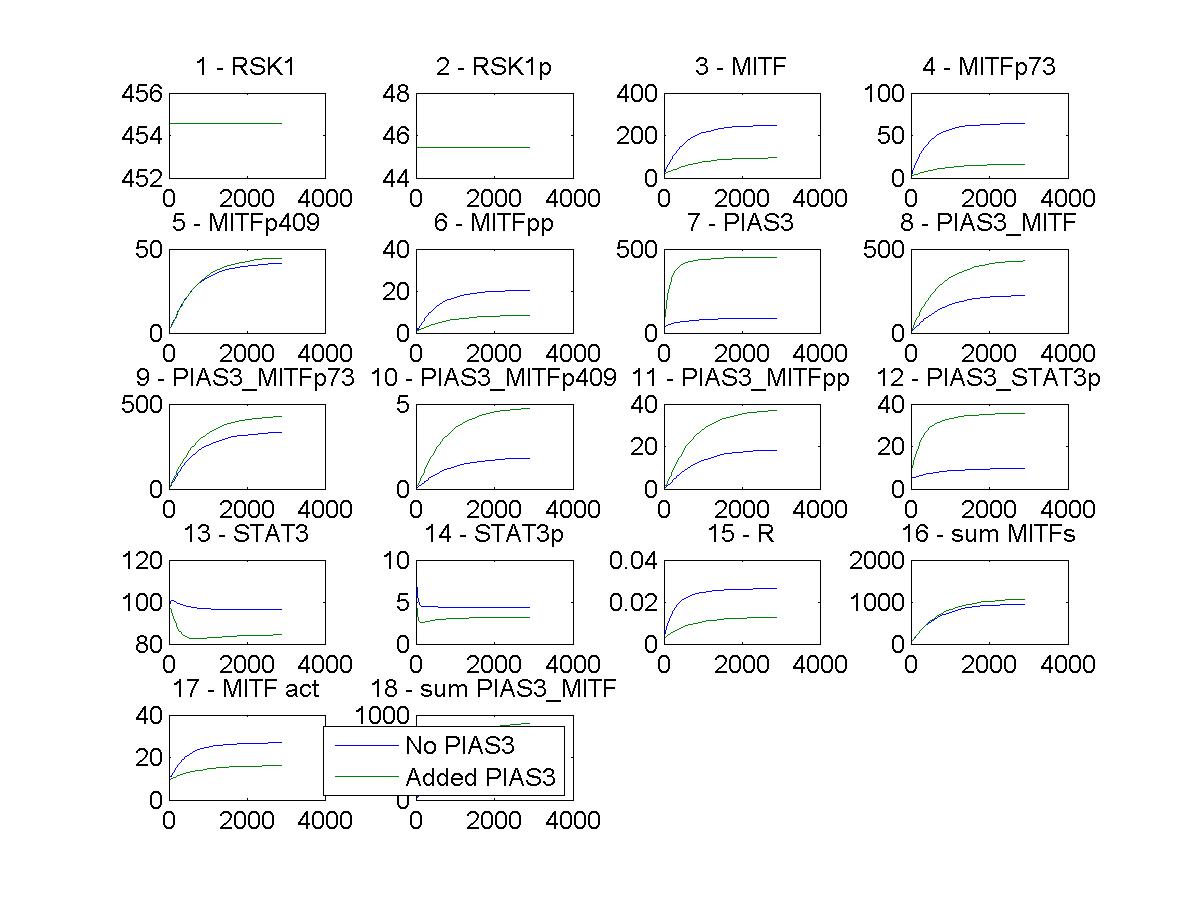

Supplement: Additional file 1 — A zip-file containing temporal plots for all variables for all simulated experiments with default parameters. The figures are in jpg-format. [file 1752-0509-6-11-S1.ZIP › simulationExperiment13.jpg]

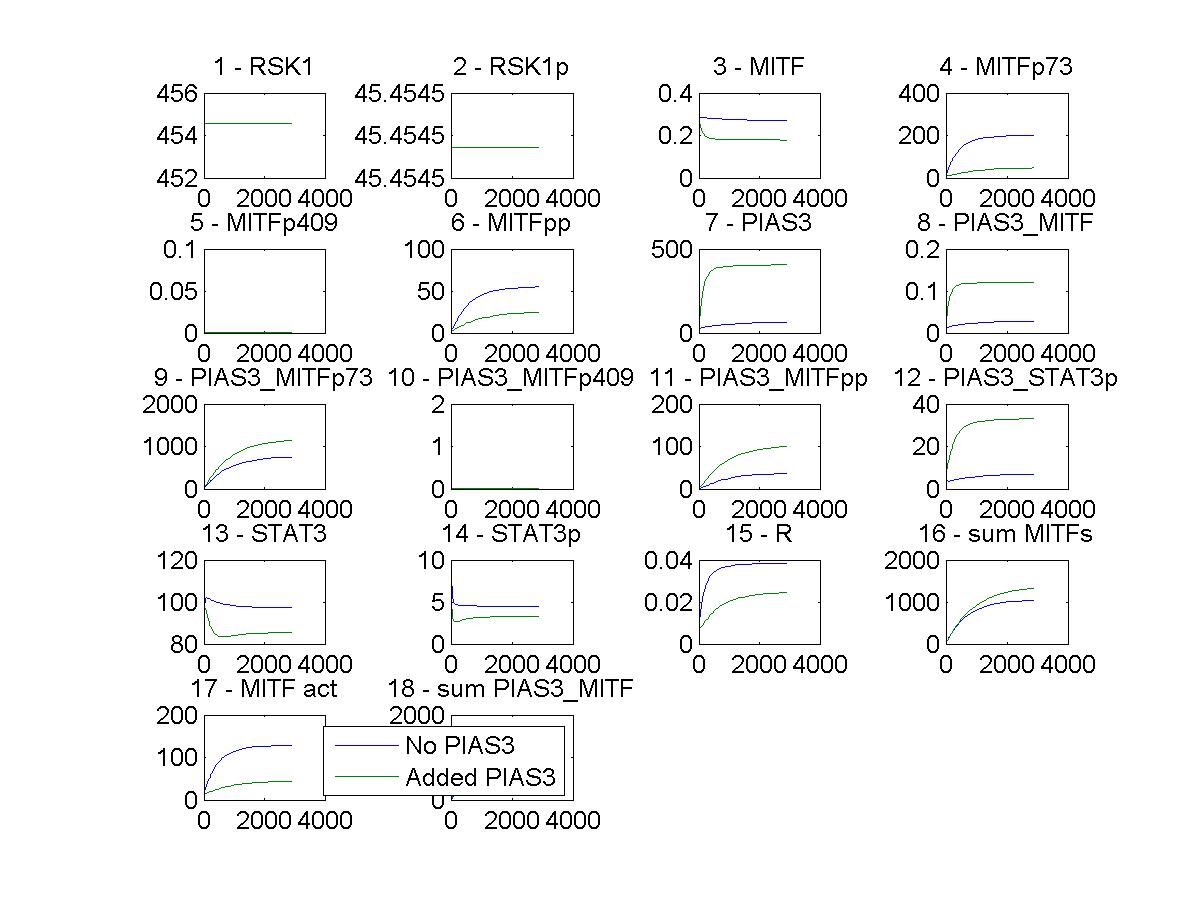

Supplement: Additional file 1 — A zip-file containing temporal plots for all variables for all simulated experiments with default parameters. The figures are in jpg-format. [file 1752-0509-6-11-S1.ZIP › simulationExperiment14.jpg]

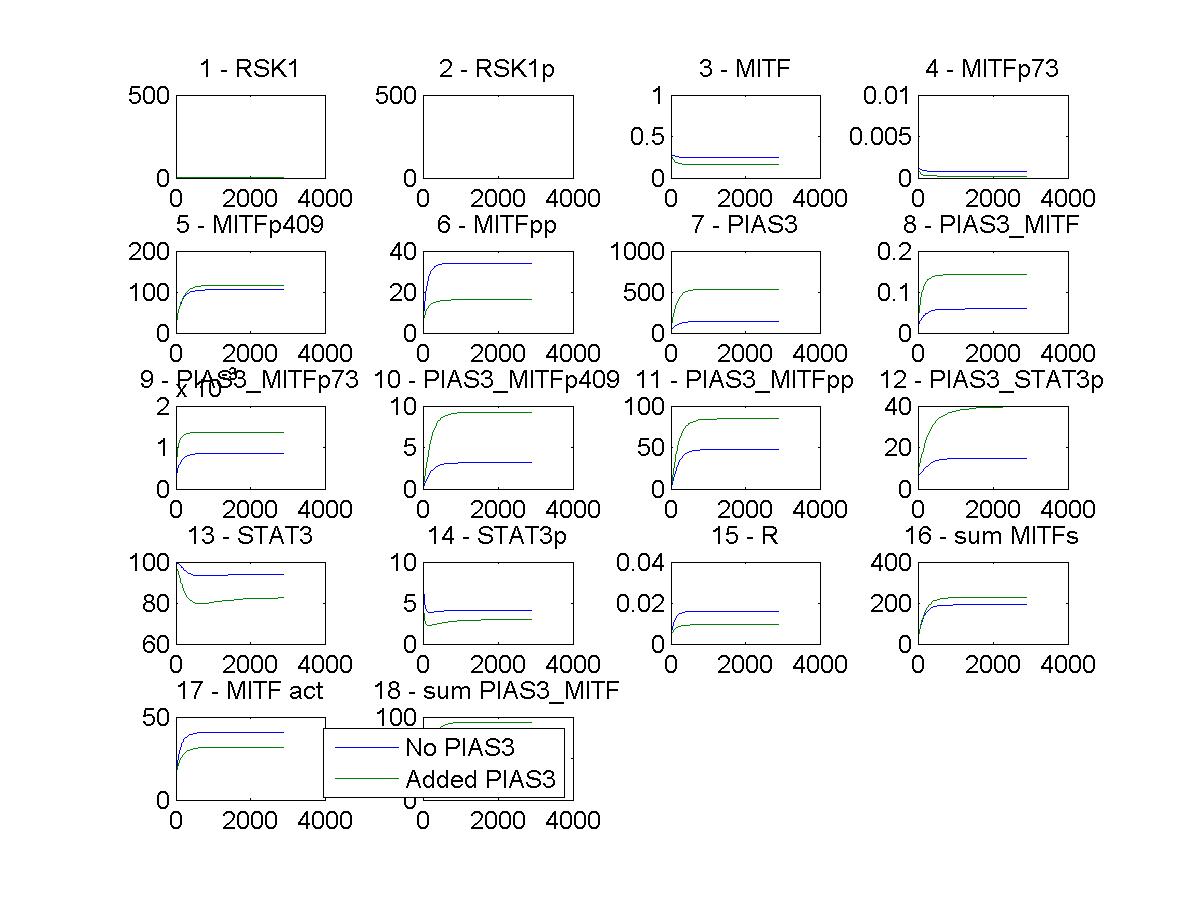

Supplement: Additional file 1 — A zip-file containing temporal plots for all variables for all simulated experiments with default parameters. The figures are in jpg-format. [file 1752-0509-6-11-S1.ZIP › simulationExperiment15.jpg]

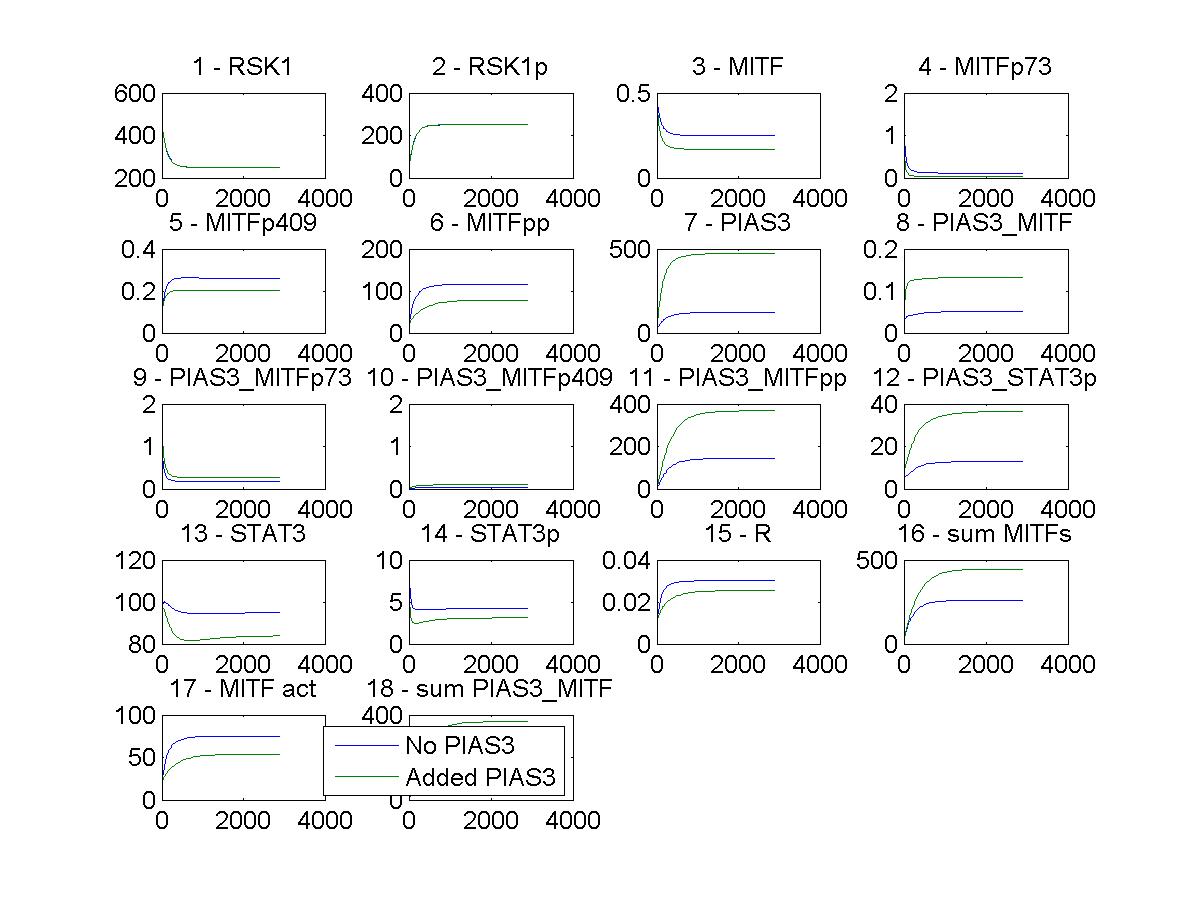

Supplement: Additional file 1 — A zip-file containing temporal plots for all variables for all simulated experiments with default parameters. The figures are in jpg-format. [file 1752-0509-6-11-S1.ZIP › simulationExperiment16.jpg]

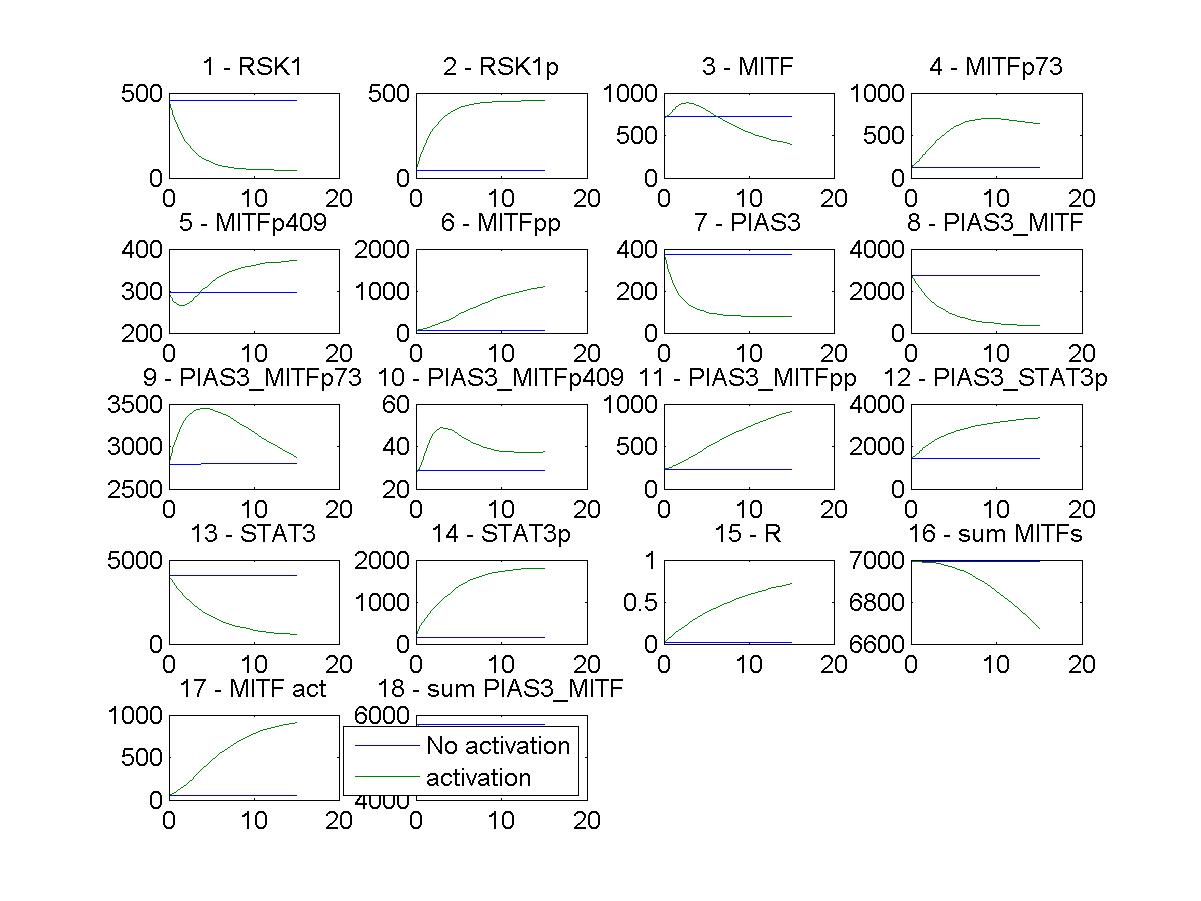

Supplement: Additional file 1 — A zip-file containing temporal plots for all variables for all simulated experiments with default parameters. The figures are in jpg-format. [file 1752-0509-6-11-S1.ZIP › simulationExperiment17.jpg]

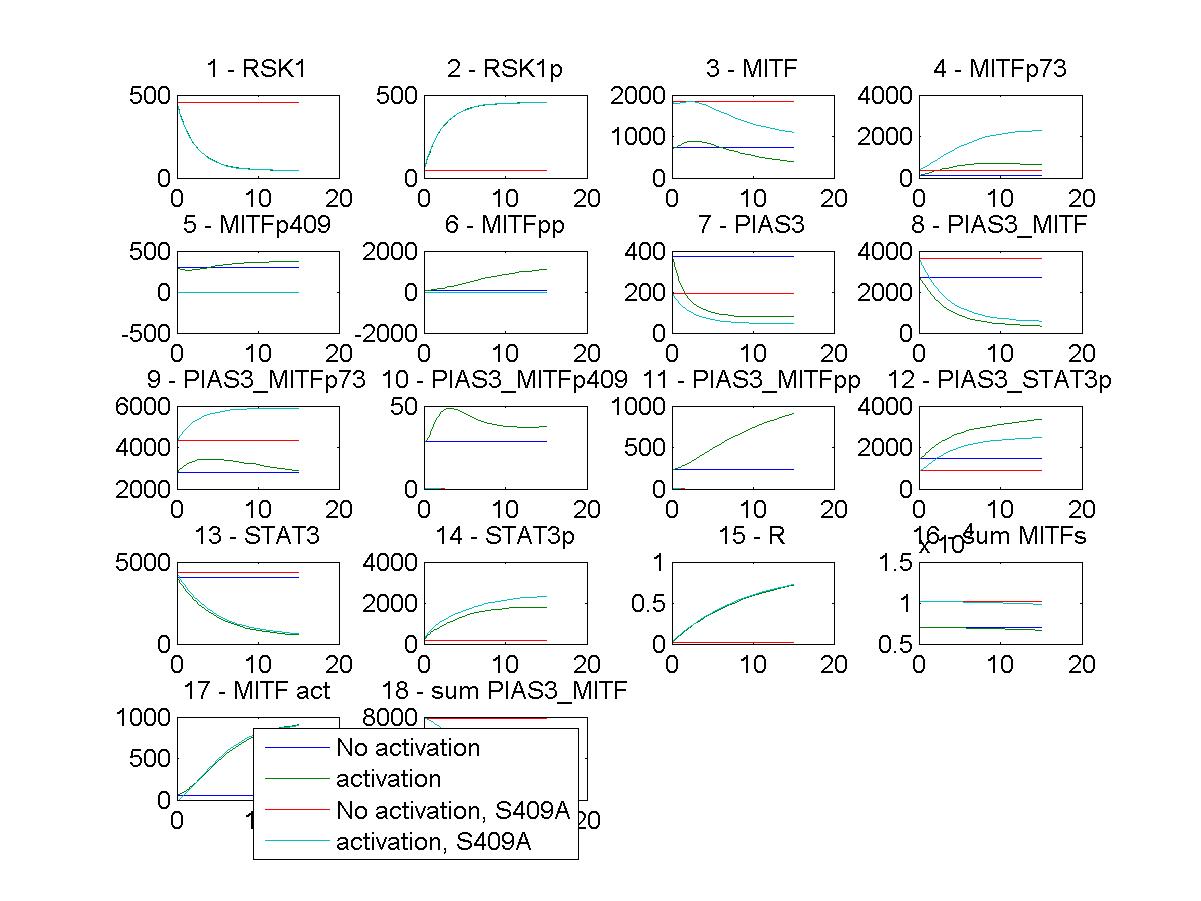

Supplement: Additional file 1 — A zip-file containing temporal plots for all variables for all simulated experiments with default parameters. The figures are in jpg-format. [file 1752-0509-6-11-S1.ZIP › simulationExperiment18.jpg]

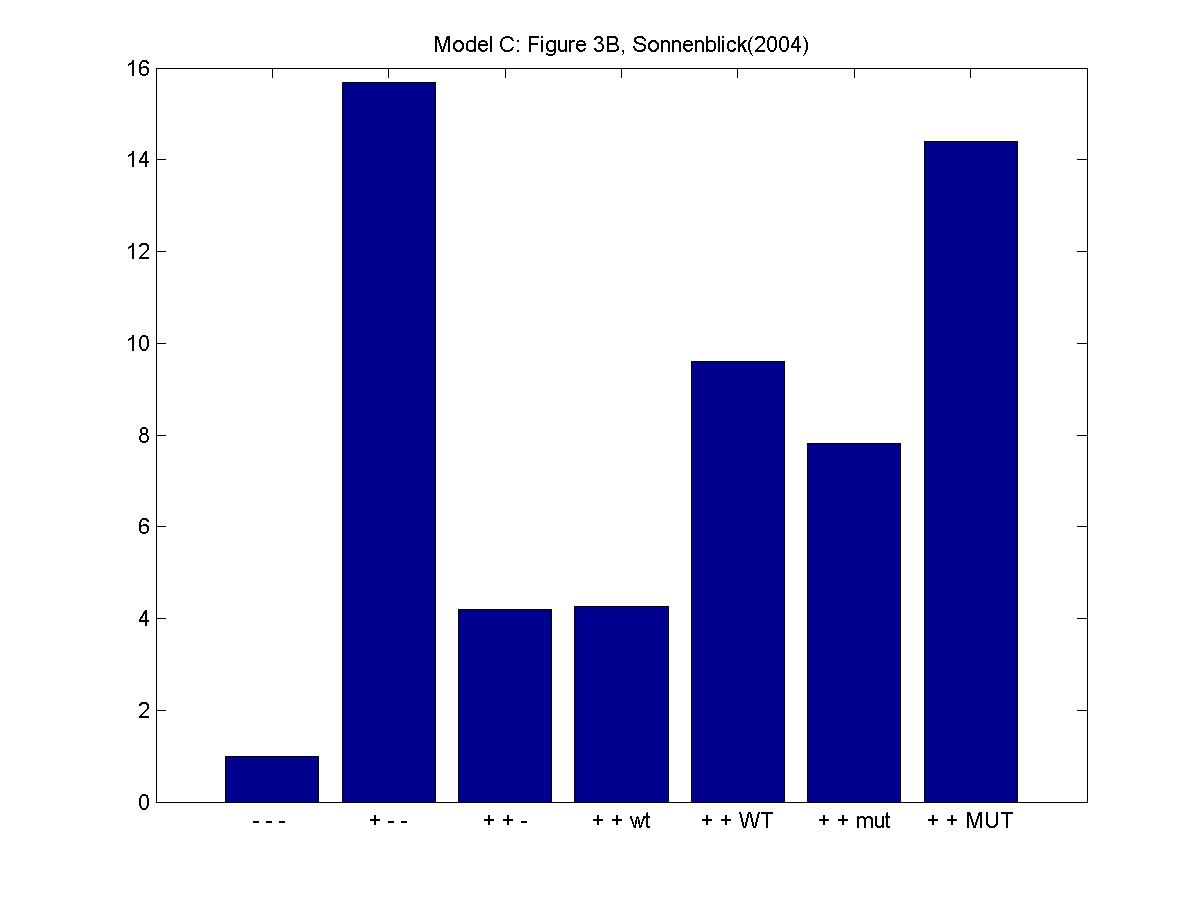

Supplement: Additional file 1 — A zip-file containing temporal plots for all variables for all simulated experiments with default parameters. The figures are in jpg-format. [file 1752-0509-6-11-S1.ZIP › simulationExperiment19-24.jpg]

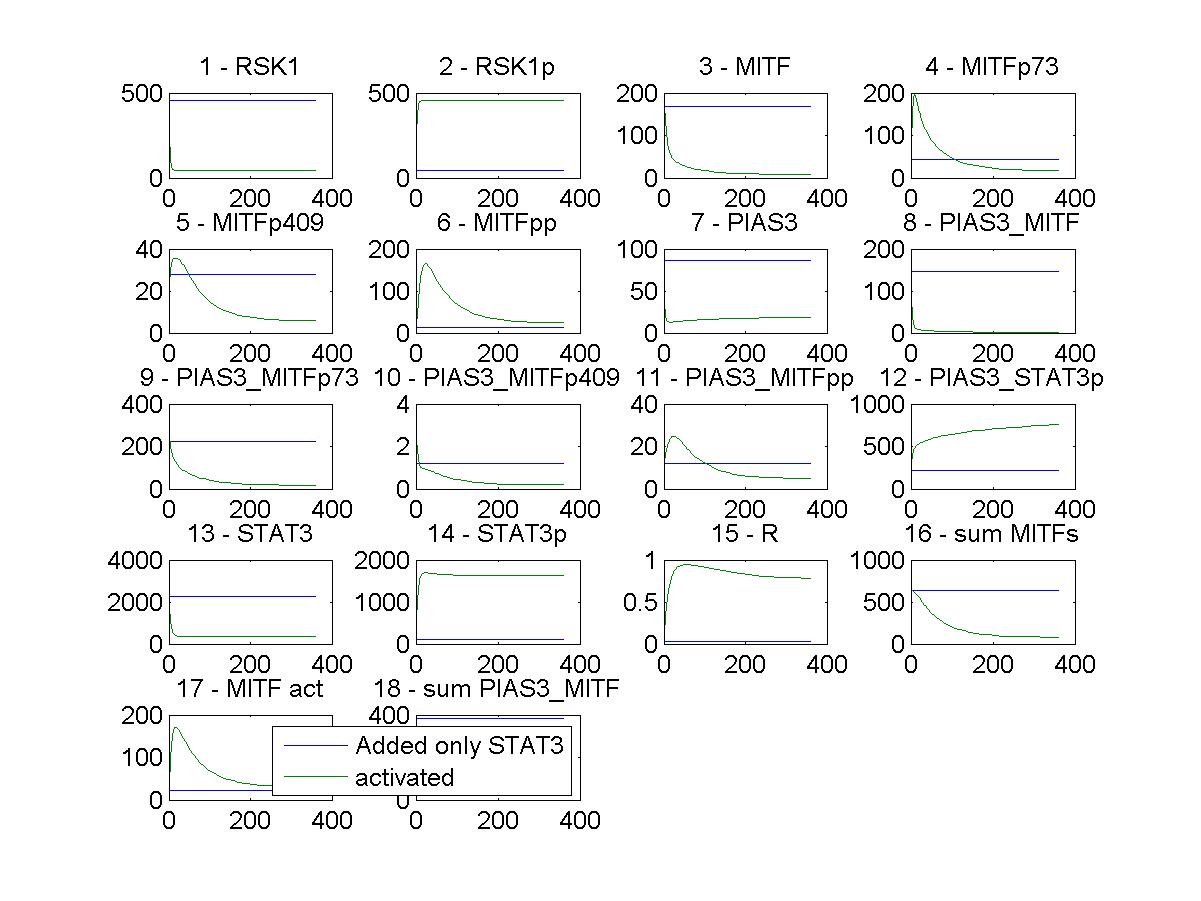

Supplement: Additional file 1 — A zip-file containing temporal plots for all variables for all simulated experiments with default parameters. The figures are in jpg-format. [file 1752-0509-6-11-S1.ZIP › simulationExperiment19.jpg]

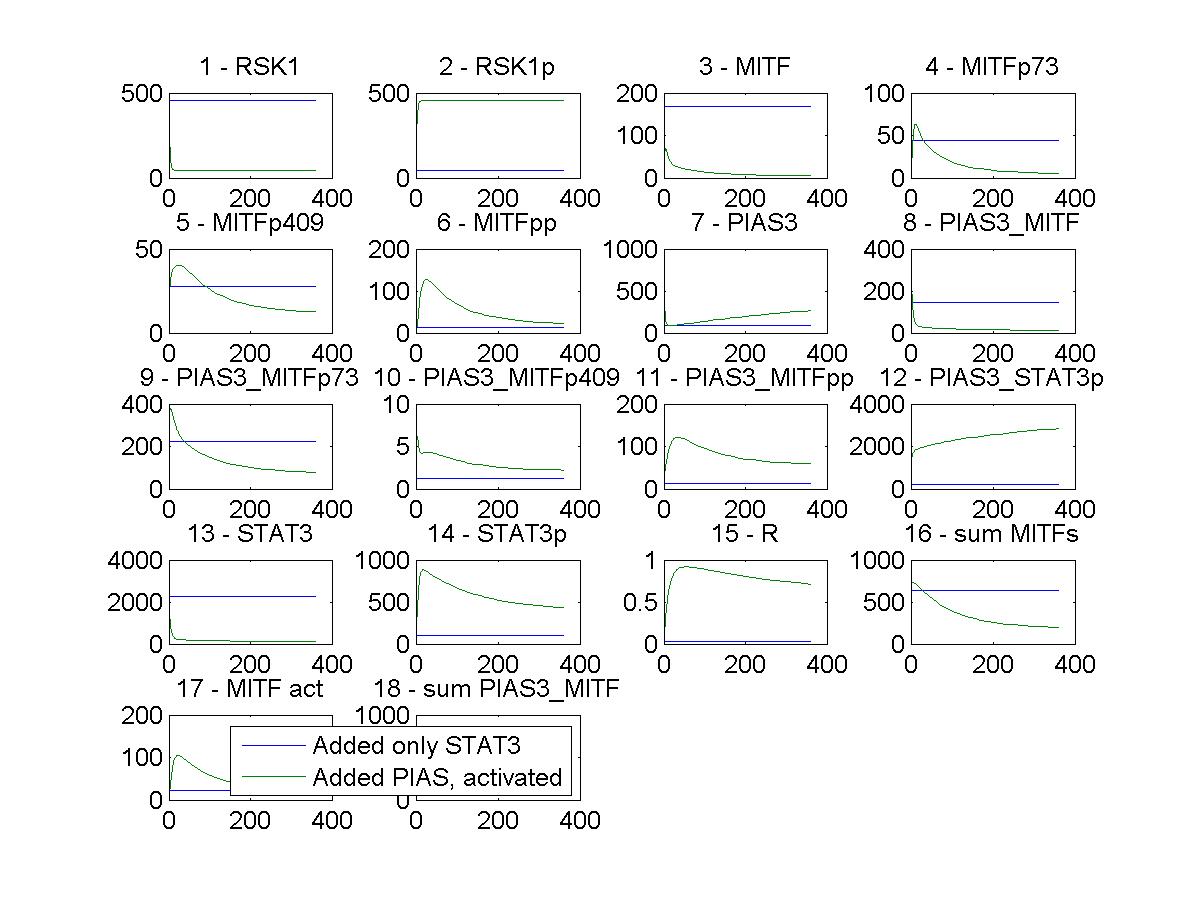

Supplement: Additional file 1 — A zip-file containing temporal plots for all variables for all simulated experiments with default parameters. The figures are in jpg-format. [file 1752-0509-6-11-S1.ZIP › simulationExperiment20.jpg]

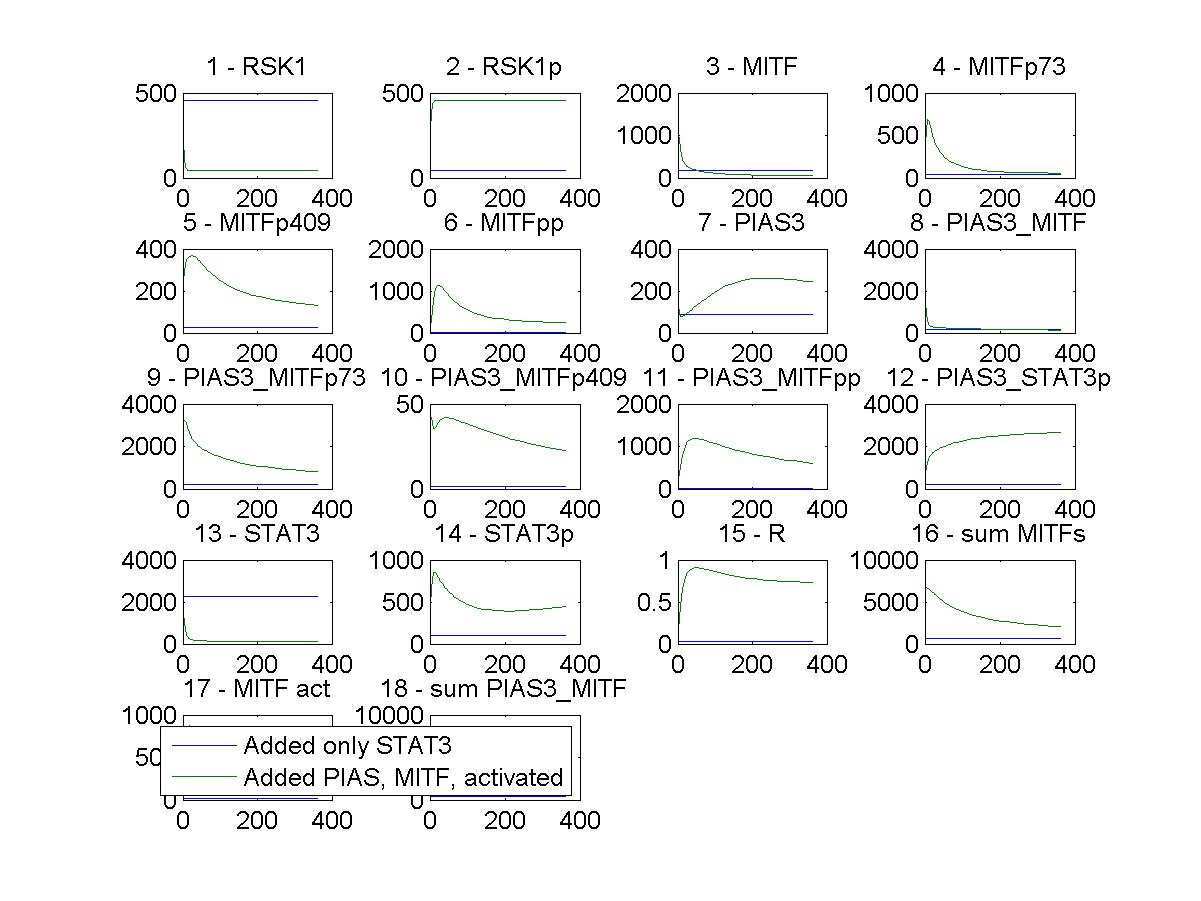

Supplement: Additional file 1 — A zip-file containing temporal plots for all variables for all simulated experiments with default parameters. The figures are in jpg-format. [file 1752-0509-6-11-S1.ZIP › simulationExperiment21.jpg]

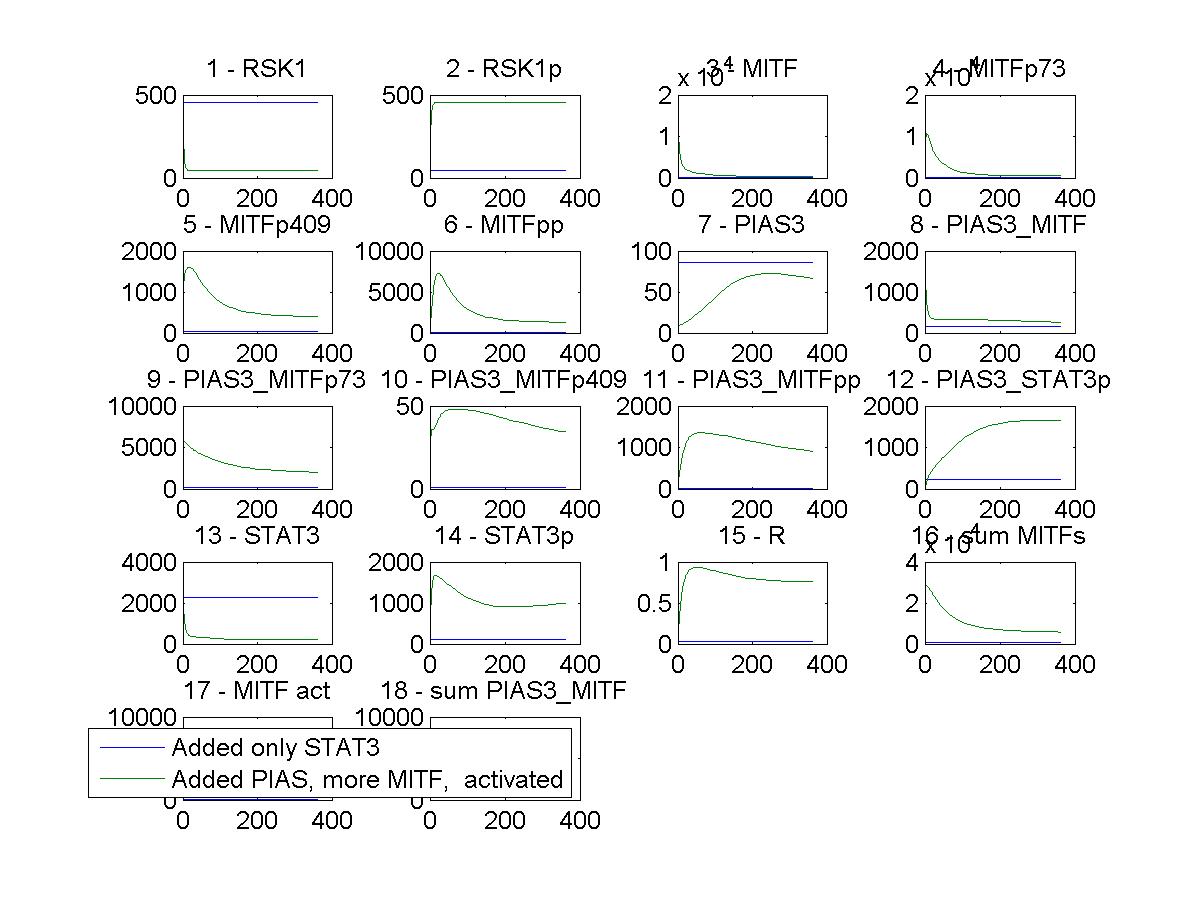

Supplement: Additional file 1 — A zip-file containing temporal plots for all variables for all simulated experiments with default parameters. The figures are in jpg-format. [file 1752-0509-6-11-S1.ZIP › simulationExperiment22.jpg]

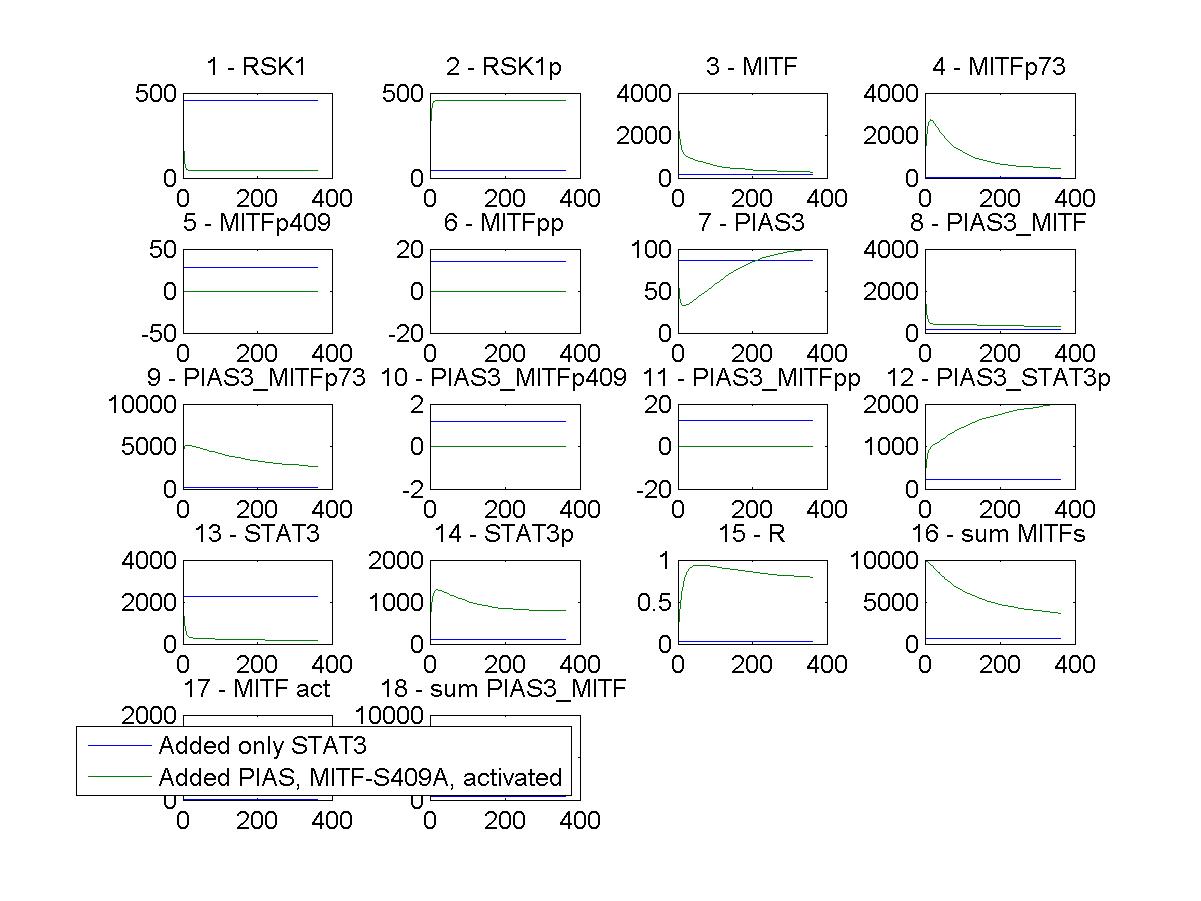

Supplement: Additional file 1 — A zip-file containing temporal plots for all variables for all simulated experiments with default parameters. The figures are in jpg-format. [file 1752-0509-6-11-S1.ZIP › simulationExperiment23.jpg]

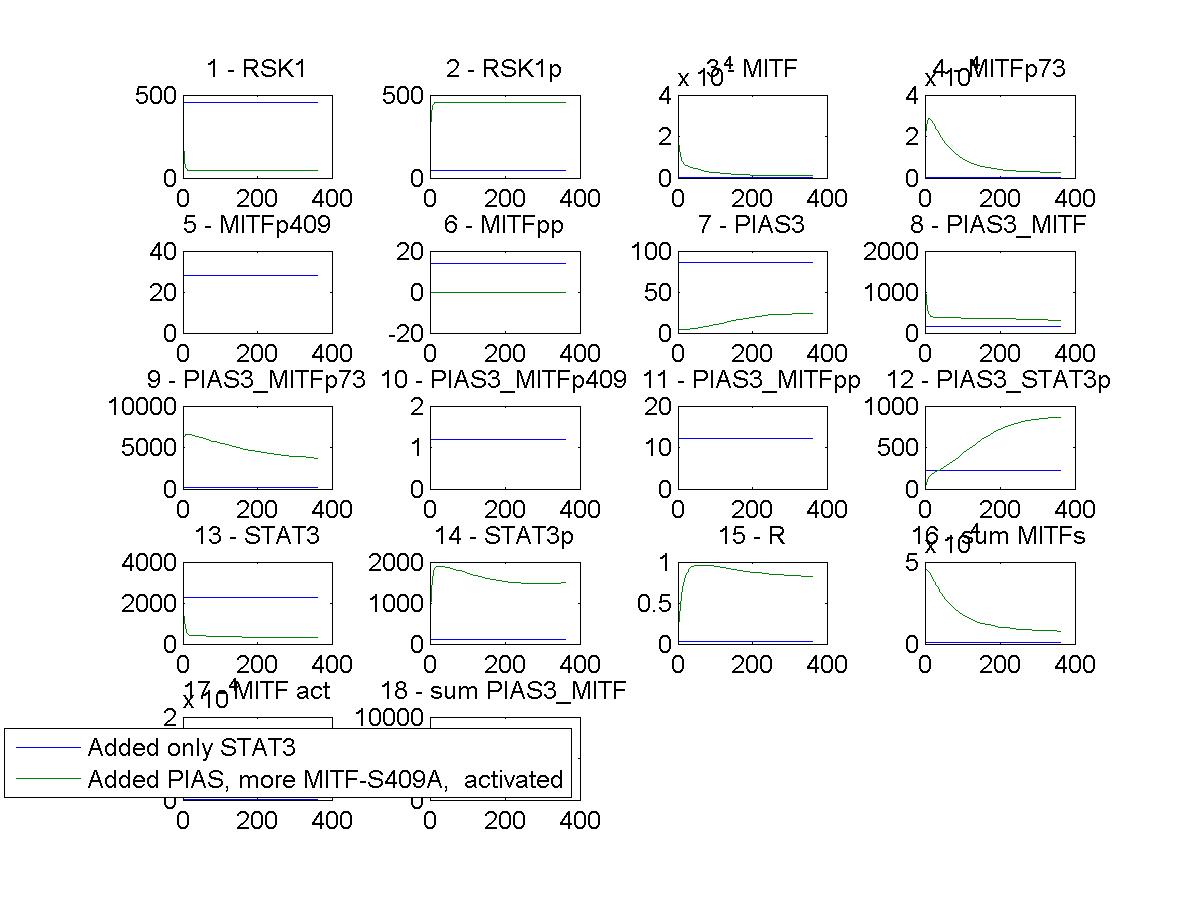

Supplement: Additional file 1 — A zip-file containing temporal plots for all variables for all simulated experiments with default parameters. The figures are in jpg-format. [file 1752-0509-6-11-S1.ZIP › simulationExperiment24.jpg]

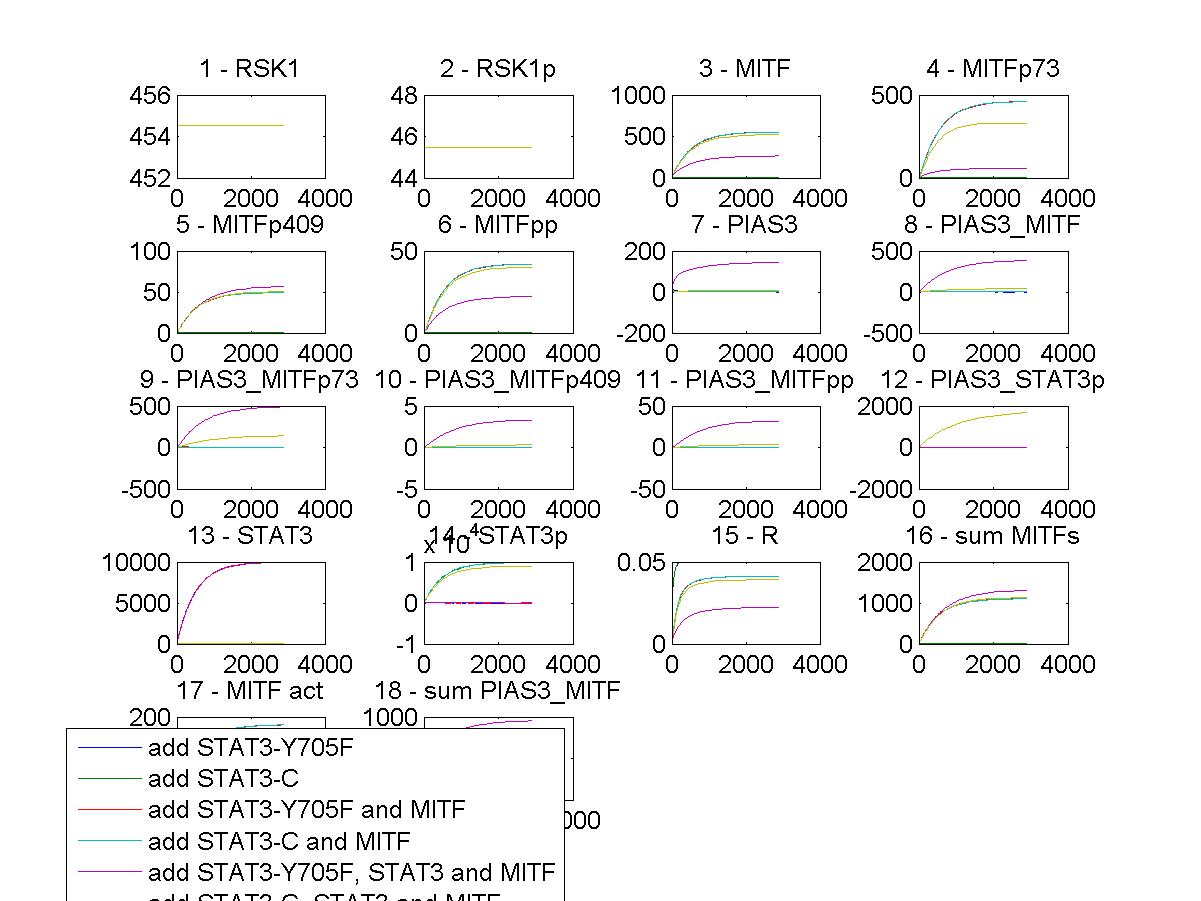

Supplement: Additional file 1 — A zip-file containing temporal plots for all variables for all simulated experiments with default parameters. The figures are in jpg-format. [file 1752-0509-6-11-S1.ZIP › simulationExperiment25.jpg]
